# Synergistic Gene Immunotherapy for Lung Cancer via Targeted Nanomedicine Restoring Genetic Tumor Suppression and Activating STING Pathway

**DOI:** 10.1021/acsnano.5c20264

**Published:** 2026-02-12

**Authors:** Weiyu Chen, Xinjie Zheng, Yuan Wu, Yiming Xu, Hangqi Ni, Peng Xiao, Weibo Cai, Kai Wang

**Affiliations:** † Department of Respiratory and Critical Care Medicine, Center for Oncology Medicine, the Fourth Affiliated Hospital of School of Medicine, and International School of Medicine, International Institutes of Medicine, 12377 Zhejiang University, Yiwu 322000, China; ‡ Zhejiang Key Laboratory of Precision Diagnosis and Treatment for Lung Cancer, Yiwu 322000, China; § College of Jiyang, Zhejiang A&F University, Zhuji 311800, China; ∥ Department of Gastroenterology, Sir Run Run Shaw Hospital, Zhejiang University School of Medicine, Hangzhou 310016, China; ⊥ Departments of Radiology and Medical Physics, 5228 University of Wisconsin-Madison, Madison, Wisconsin 53705, United States

**Keywords:** gene immunotherapy, tumor suppressor gene, STING agonist, layered double hydroxides, tumor-targeted delivery

## Abstract

Lung cancer, particularly non-small cell lung cancer (NSCLC), presents significant therapeutic challenges due to its high mortality and complex pathogenesis. General strategies, including chemotherapy, immunotherapy, and even novel gene therapy, fail to provide comprehensive inhibition against NSCLC individually. Here, a novel gene-immunotherapeutic nanomedicine, pTMEM163/cGAMP@cRGD-BSA/LDHs (TGR-BLDHs), was developed by employing cyclic Arg-Gly-Asp (cRGD)-modified bovine serum albumin/layered double hydroxide (BSA-LDH) nanoparticles for targeted delivery of TMEM163, a newly identified tumor suppressor gene (TSG) of NSCLC and cGAS/STING agonist (cGAMP). TGR-BLDHs exhibited highly specific NSCLC tumor suppression via desirable tumor-targeted TSG gene therapy. Meanwhile, TGR-BLDHs successfully evoked potent antitumor effects by activating the cGAS/STING pathway in both antigen-presenting and cancerous cells, eventually inhibiting tumor progression in vivo. The current study highlighted the potential of TGR-BLDHs for effective gene immunotherapy against NSCLC with desirable tumor specificity and biocompatibility, offering a promising gene-immunotherapeutic strategy for NSCLC.

## Graphical abstract

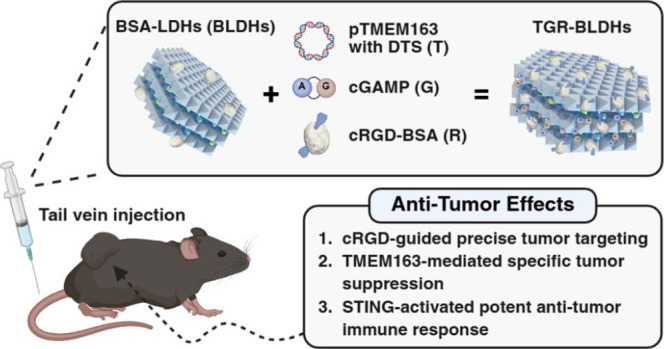

## Introduction

Lung cancer remains one of the most common and deadly malignancies, continuing to be the leading cause of cancer-related mortality worldwide, with the fastest-growing incidence and a dismal five-year survival rate of less than 20%.^1,2^ Non-small cell lung cancer (NSCLC) accounts for about 85% of lung cancer cases, including lung squamous cell carcinoma (LUSC), lung adenocarcinoma, and lung large cell carcinoma.^3,4^ According to lung cancer staging, treatments for NSCLC vary, ranging from surgery or chemotherapy to targeted therapy or immunotherapy.^5^ Despite the significant advancements in therapeutic strategies, issues such as drug resistance and postoperative recurrence persist.^6,7^ Particularly concerning are the significant side effects stemming from the shortage of drug specificity. There is a pressing need to develop more potent and precise therapeutic approaches.

One promising strategy involves tumor suppressor genes (TSGs), which play a crucial role in limiting tumorigenesis and substantially influencing therapeutic outcomes.^8^ Typical TSG-based gene therapy has been evaluated in suppressing various tumor types, such as TP53 gene editing via a viral vector.^9^ Notably, the tissue-specific TSG would potentially enhance therapeutic outcomes compared to broad-spectrum TSG, particularly lowering off-target effects such as arresting the normal cell cycle via off-target TP53 overexpression. According to our bioinformatics analysis, transmembrane 163 (TMEM163), a zinc-binding protein, was identified, and its high expression in NSCLC was strongly associated with a lower risk of death (Figure 1A,B). Thus, it is vital to evaluate the role of TMEM163 in tumorigenesis and its potential as a TSG for NSCLC gene therapy.

**Figure 1:**
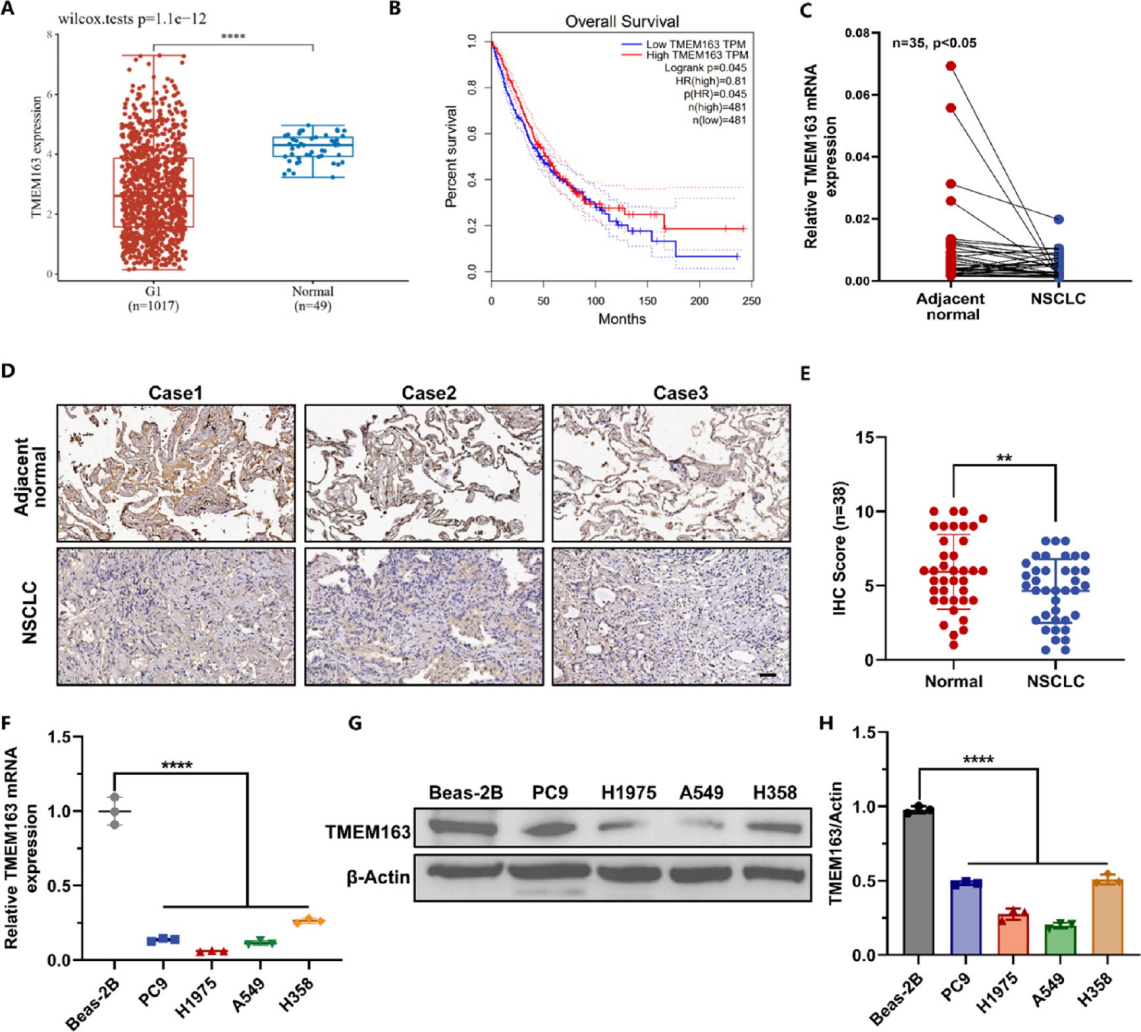
TMEM163 is downregulated in NSCLC and associated with optimistic prognosis. (A) TMEM163 expression in NSCLC cohorts and normal tissues from the TCGA database. (B) Correlation of overall survival of patients with TMEM163 expression. (C) Relative expression level of TMEM163 mRNA from patient’s tumor compared with paired adjacent normal tissues (*n* = 35). Scale bar, 50 μm. (D,E) Immunohistochemistry analysis for expression of TMEM163 in NSCLC tissues compared with paired adjacent normal tissues (*n* = 40). (F) Relative gene expression of TMEM163 in normal human lung epithelia cells and NSCLC cell lines. (G,H) Relative protein levels of TMEM163 in normal human lung epithelia cells and NSCLC cell lines. Statistical significance was determined using a two-tailed Student’s *t*-test. Data are presented as mean ± SEM, *p < 0.05; **p < 0.01; ****p < 0.0001.

Meanwhile, as an excellent TSG gene carrier, plasmid DNA (pDNA) has excellent stability and is easy to preserve.^10^ It is notable that incorporating a DNA targeting sequence (DTS), short conserved motifs recognized by specific transcription factors, could enhance the nucleus entry of pDNA.^11^ By employing DTS in TSG pDNA, such as the TMEM163 plasmid (pTMEM163), it would potentially promote gene expression, achieving desirable therapeutic efficiency. However, the clinical translation of TSG-based therapeutics remains severely hindered by several intrinsic limitations. First, most naked plasmid DNAs suffer from rapid enzymatic degradation and poor physicochemical stability in the bloodstream, resulting in an extremely low circulation half-life and minimal accumulation at tumor sites. The negatively charged and hydrophilic nature of plasmids also prevents their efficient interaction with cell membranes, further diminishing the intracellular delivery efficiency. Second, even when encapsulated by conventional vectors such as cationic liposomes or polymeric nanoparticles, these systems often face serious challenges, including serum-induced aggregation, rapid opsonization, and nonspecific hepatic uptake, leading to inefficient tumor accumulation and off-target toxicity.^12^ Moreover, the excessive surface charge required for complexation can cause cytotoxicity and trigger undesired immune responses. The lack of delivery vehicles severely limits the TSG pDNA application.

Coincidentally, nanomaterials have emerged as desirable carriers for functional gene delivery and genetic therapy due to their advanced physicochemical properties.^13,14^ Among all, compared with conventional nanocarriers such as cationic liposomes, polymeric vectors (e.g., PEI), and inorganic oxide nanoparticles, layered double hydroxides (LDHs) feature positively charged lamellar structures and serve as powerful platforms for delivering therapeutic agents (e.g., nucleic acid), given their excellent features, such as intrinsic ability for multicargo loading, pH-dependent biodegradability, easy surface modification, inherent biocompatibility, and superior chemical stability.^15–18^ Especially, LDHs are able to deliver biomacromolecules like pDNA and successfully mediate the overexpression of functional protein.^19^ Additionally, targeted nanomaterials are engineered to precisely deliver therapeutic agents to pathological cells, thereby mitigating potential off-target effects on healthy tissues. Among the various targeting ligands developed to date, cyclic Arg-Gly-Asp (cRGD) peptide, an αvβ3 integrin-targeting ligand, is expressed homogeneously distributed in lung carcinoma at a high level and rarely in normal tissues,^20,21^ making it a widely investigated and potent agent for improving drug targeting precision and therapeutic efficacy.^22–24^ Therefore, successful surface modifications of cRGD on LDHs would further enhance the therapeutic specificity of NSCLC-specific TSG (e.g., pTMEM163) gene therapy.

Notably, nanomaterials will be inevitably internalized by antigen-presenting cells (APCs) during circulation, which may decrease the therapeutic efficiency to some extent. Most existing gene delivery platforms are designed for single-agent delivery and thus fail to achieve the coordinated modulation of tumor immunity. In the context of NSCLC, where immune evasion and tumor heterogeneity critically limit therapeutic efficacy, monogenic restoration alone is insufficient to elicit durable antitumor responses. To better use this situation, incorporating immune-activating ligands would help modulate the immune response favorably. The STING pathway, crucial for antitumor immunity, detects intracellular DNA and triggers the synthesis of type I interferons and various inflammatory mediators, thus enhancing the immune system’s ability to identify and eradicate tumor cells.^25^ Cyclic-di-GMP-AMP (cGAMP) acts as a secondary messenger and potent activator for the STING pathway, thereby enhancing the immunogenic properties of cancer cells. By incorporation of nanomaterials, the stability and delivery efficiency of cGAMP could be strongly enhanced, significantly prolonging circulation time and improving tumor accumulation. In light of these advancements, gene-immunotherapy, which combines gene therapy with immune modulation, emerges as a compelling approach. This strategy leverages the strengths of both modalities to target disparate mechanisms and elicit synergistic antitumor outcomes.

Herein, we developed a novel nanomedicine, pTMEM163/cGAMP@cRGD-BDA/LDHs (TGR-BLDHs), for targeted gene immunotherapy in NSCLC by codelivering pTMEM163 (coding with DTS sequence) and cGAMP. We first evaluated that pTMEM163 effectively inhibited cancer cell proliferation, migration, and invasion through the p38 MAPK signaling pathway, emphasizing its role in cancer suppression. Then, the pTMEM163 and BLDH complex (T-BLDH) was designed to target and efficiently introduce therapeutic genes into cancer cells. Notably, additions of cRGD and cGAMP further enhanced delivery efficiency and boosted the antitumor immune response mediated by T-BLDHs. Most importantly, TGR-BLDHs successfully triggered potent antitumor immune activity by elevating the number and function of tumor-infiltrating lymphocytes, including CD8+ T cells and dendritic cells (DCs), and by activating the cGAS/STING pathway in APCs. Thus, TGR-BLDHs represented a promising, efficient, and low-toxicity gene immunotherapy with enhanced tumor targeting and immune activation (Scheme 1).

**Scheme 1:**
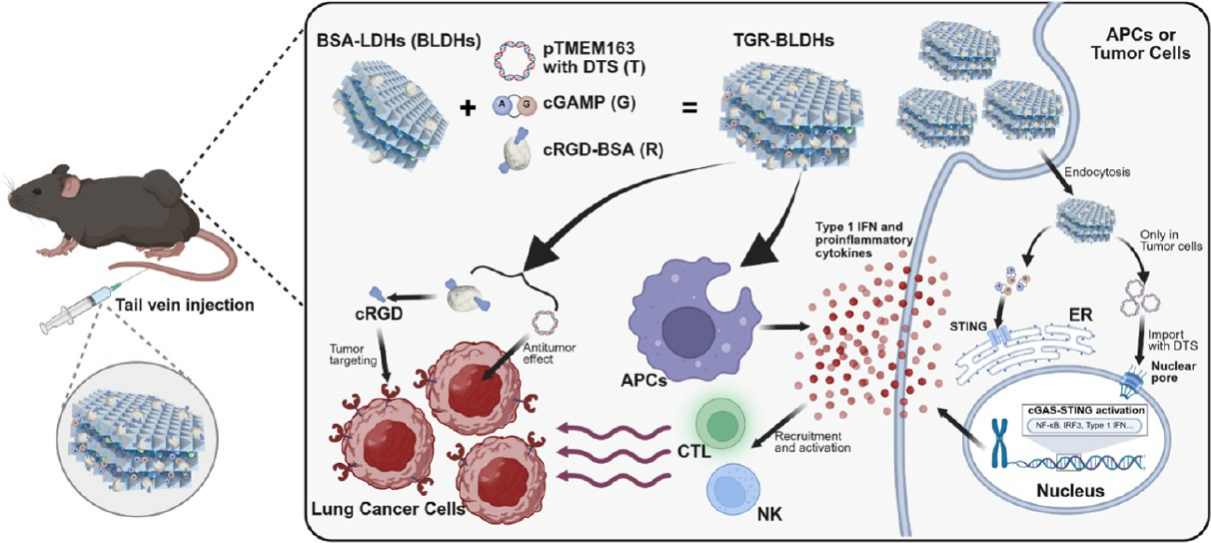
Schematic of the Therapeutic Process of TGR-BLDHs

## Results and Discussion

### TMEM163 Is Downregulated in NSCLC and Associated with an Optimistic Prognosis

According to the data sets from the TCGA database, a lower expression level of TMEM163 was found in three NSCLC cohorts (Figure 1A) compared with the normal tissues, which was associated with poor outcomes (Figure 1B). For further evaluation, TMEM163 in NSCLC clinical samples was examined as well. Figure 1C shows that TMEM163 mRNA was downregulated in lung malignant tissues. Moreover, TMEM163 significantly decreased in NSCLC tissues compared with paired adjacent normal lung tissues, as assessed by immunohistochemistry (lHC) assay (Figure 1D,E). Similarly, RT-qPCR (Figure 1F) and Western blot (Figure 1G,H) indicated that TMEM163 mRNA and protein levels were lower in NSCLC cells than in normal lung cells, for instance, BEAS-2B cells. Thus, TMEM163 is downregulated in lung cancer tissues and is strongly associated with favorable outcomes.

### TMEM163 Functions as a Potent TSG and Impairs Lung Cancer Cell Growth, Proliferation, and Migration

To validate its function, TMEM163 was overexpressed in NSCLC cell lines (Figure 2A). CCK-8 assays revealed that cell growth decreased remarkably upon TMEM163 overexpression, reducing the growth rate to 57.9% and 56.3% in A549 and H1975 cells, respectively (Figure 2B). Furthermore, EdU staining demonstrated that TMEM163 overexpression inhibited cell proliferation in A549 and H1975 cells, reducing it to approximately 44.8% or even lower (15.4%; Figure 2C,D). The impact on migration capability was then assessed, showing that TMEM163 overexpression significantly inhibited wound-healing capacity (reducing to approximately 23.3% and 30.3% in 24 h) (Figure 2E,F) and cellular migration (reducing to approximately 36.1% and 12.3%; Figure 2G) of A549 and H1975 cells. Overexpression of TMEM163 also significantly inhibited the cells’ ability to form colonies in 2-D plates, reducing colony numbers from 207 to 134 and 156 to 71 in A549 and H1975 cells (Figure 2H). Additionally, TMEM163 did not affect the cell cycle and apoptosis (Figure S1A,B).

**Figure 2:**
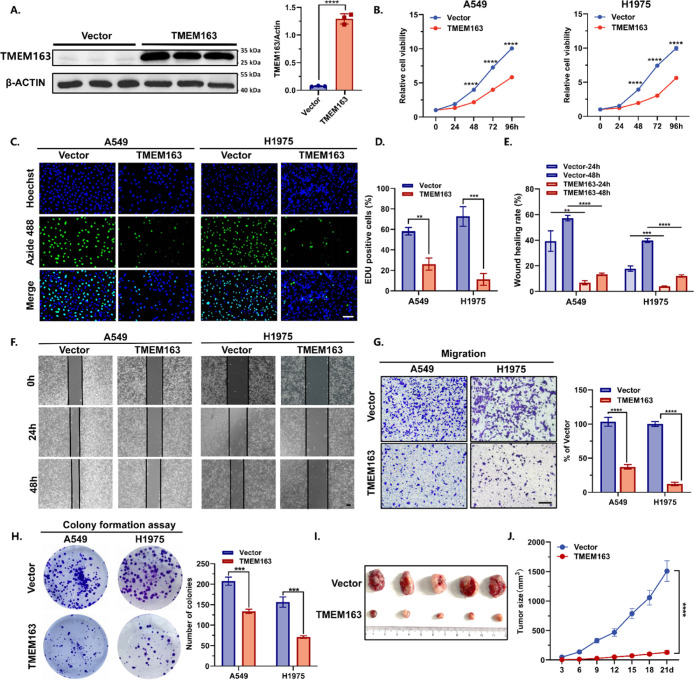
TMEM163 impairs lung cancer cell proliferation, growth, invasion, and migration in vitro. (A) Western blot analysis confirming the efficiency of TMEM163 overexpression in A549 cells (*n* = 3). (B) Cell viability assessment of A549 and H1975 cells overexpressing TMEM163 using CCK-8 assays (*n* = 3). (C) Representative images depicting EdU staining in A549 and H1975 cells with TMEM163 overexpression (*n* = 3). Scale bar, 100 μm. (D,E) Quantification of the ratio of EdU-positive cells to total Hoechst-positive cells (D) and the wound-healing rate (E) using ImageJ software. (F) Representative images from the wound-healing assay of A549 and H1975 cells with TMEM163 overexpression. Images were acquired at 0, 24 h, and 48 h postscratching (*n* = 3). Scale bar, 200 μm. (G) Illustrative images showcasing the Transwell migration of A549 and H1975 cells overexpressing TMEM163 (*n* = 3). Scale bar, 100 μm. (H) Illustrative images of the colony formation assay in A549 and H1975 cells overexpressing TMEM163 (*n* = 3). (I,J) Photographic representation of harvested subcutaneous tumors (I) at the time of sacrifice. Tumor growth curves (J) derived from subcutaneous xenograft models with stable TMEM163 overexpression and negative control (*n* = 5). Statistical significance was determined using a two-tailed Student’s *t*-test. Data are presented as mean ± SEM, *p < 0.05; **p < 0.01; ****p < 0.0001.

To further investigate the anticancer effect of TMEM163 in vivo, subcutaneous xenograft models were established using stable TMEM163 overexpression or normal LLC cells, respectively. In alignment with the in vitro results, tumors derived from TMEM163-overexpressing cells exhibited significantly reduced growth rates compared to the control group (Figure 2I). More importantly, the stable expression of TMEM163 resulted in almost no tumor growth by the third week. Moreover, the average tumor volume decreased remarkably compared to that of the control group (Figure 2J). Overall, these gain-of-function assays confirm that TMEM163 inhibits NSCLC in vitro and in vivo.

### TMEM163 Overexpression Constrains NSCLC Cells by Attenuating p38 MAPK Signaling

The molecular mechanisms underlying the TMEM163-induced antitumor effect were further investigated. According to RNA-sequencing, TMEM163 overexpression resulted in 568 genes being upregulated and 388 genes being downregulated for more than 2-fold (Figure S2A). According to the Kyoto Encyclopedia of Genes and Genomes (KEGG) database, the most significantly altered pathways were associated with the MAPK signaling pathway, cytokine–cytokine receptor interaction, NF–kappa B signaling pathway, and others (Figure 3A). Consistent with RNaseq and KEGG analysis, the Western blot assessments confirmed the changes in various signaling pathways, especially attenuation in the p38 MAPK pathway after TMEM163 treatments (Figure 3B–D). Additionally, by analyzing molecular interaction forces, the MAPK14-TMEM163 score was −529, with scores below −400 indicating strong interaction (Figure S3A). In line with this finding, p38 MAPK phosphorylation was observed to be diminished in response to TMEM163 overexpression (Figure 3C,D).

**Figure 3:**
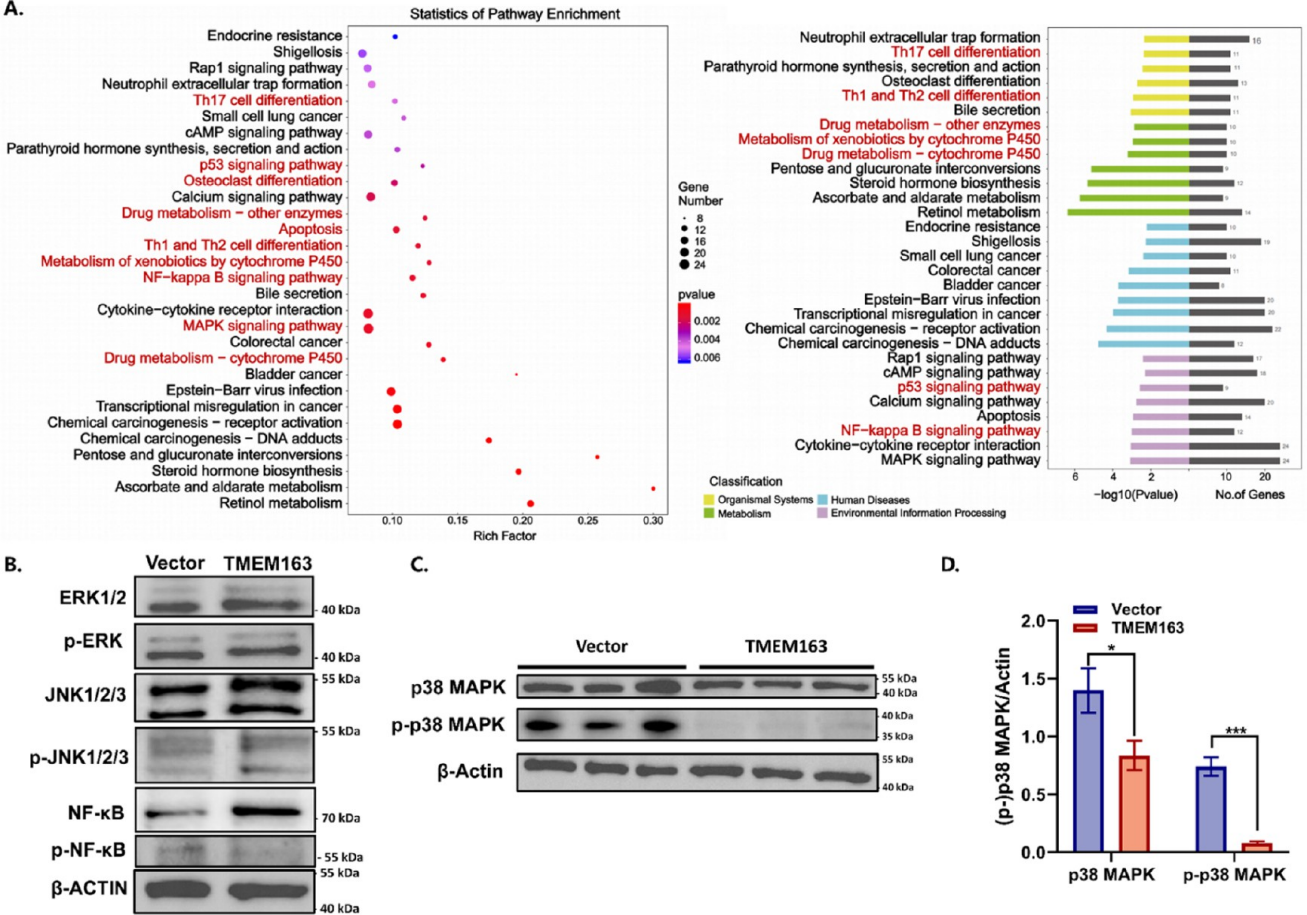
TMEM163 overexpression attenuates p38 MAPK signaling. (A) KEGG pathway enrichment analysis. (B) Various signaling pathways were examined through Western blot analysis. (C,D) Relative protein level of p38 MAPK and p-p38 MAPK in TMEM163-overexpression samples (*n* = 3). Statistical significance was determined using a two-tailed Student’s *t*-test. Data are presented as mean ± SEM, *p < 0.05; **p < 0.01; ****p < 0.0001.

The notable antitumor effects of TMEM163 prompted the consideration of gene therapy to restore its normal function in tumors. However, the efficiency of monogene therapy is generally unsatisfactory and requires reinforcement from other combined therapies. Notably, drug metabolism was accelerated after TMEM163 treatment, which may be caused by tumors’ stress responses and casts doubt on combined chemotherapy (Figure 3A). In contrast, the activities of immune-related pathways were heightened. More specifically, analysis shows that TMEM163 positively correlates with STING1 (Figure S3B). In other words, cGAS in tumor cells can sense intracellular damaged DNA and activate STING after TMEM163 functioning (e.g., pTMEM163), altering the tumor immune microenvironment (TIME). These findings indicate the great potential of combining immunotherapy with TMEM163 gene therapy for tumor eradication.

### Preparation and Characterization of TGR-BLDHs

To achieve precision gene immunotherapy, a tumor-targeted multifunctional nanomedicine was designed. We adopted a stepwise loading process to minimize the interference. The pTMEM163 and the costimulator cGAMP (STING agonist) were sequentially integrated into BSA/LDHs (BLDHs) with surface modification by cRGD (Figure 4A). The BSA coating on the LDH core plays a dual role: it not only enhances biocompatibility but also provides abundant hydrophilic functional groups (−NH_2_, −COOH, and −OH) that can establish hydrogen bonding and electrostatic interactions with cGAMP.^26^ These interactions slow desorption in physiological media. Transmission electron microscopy images showed that cRGD@BSA/LDH (R-BLDH) nanocomposites loaded with pDNA and cGAMP had good dispersion and uniformity (Figure 4B,C), presenting a typical hexagonal morphology with a diameter of ∼ 191 nm characterized by the Nano Laser Particle Size Analyzer (Figure 4D). Meanwhile, TGR-BLDHs carried a negative charge at −31.4 mV (Figure 4E). The loading capacities of pTMEM163 and cGAMP in BLDHs were further determined, respectively, while the optimal mass ratio of BLDHs/pTMEM163/cGAMP was set at 40:1:1 (Figures 4F,G, and S4A). The actual encapsulation efficiencies were 95.6 ± 1.5% for pTMEM163 and 90.8 ± 2.1% for cGAMP, respectively, which demonstrated that both cargos were efficiently captured and retained by the BLDHs.

**Figure 4:**
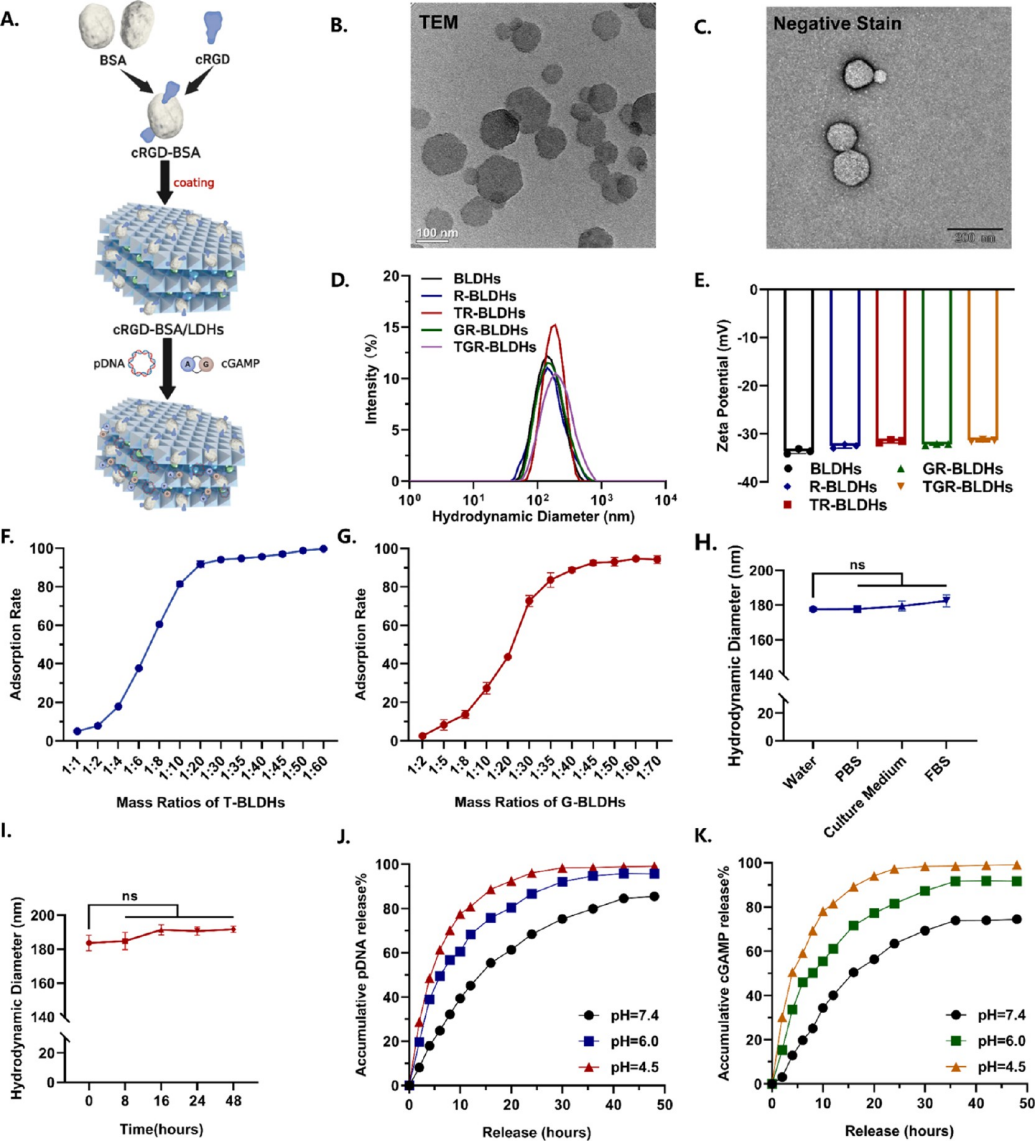
Characteristics of TGR-BLDHs loaded with pDNA and cGAMP. (A) Schematic representation of the components of the nanocomposite. (B,C) Scanning electron microscopy image of TGR-BLDHs. Scale bar, 100 and 200 nm. (D,E) Hydrodynamic size and zeta potential of different nanocomposites. (F,G) The assay of loading content of pDNA or cGAMP in BSA/LDHs (*n* = 3). (H,I) Hydrodynamic size of TGR-BLDHs in different solutions (H) and in PBS at different times (I) (*n* = 3). (J,K) The assay of release ability of pDNA and cGAMP under different pH conditions. Statistical significance was determined using a two-tailed Student’s *t*-test. Data are presented as mean ± SEM, *p < 0.05; **p < 0.01; ****p < 0.0001.

The measurement of the hydrodynamic diameter showed that TGR-BLDH nanocomposites exhibited good dispersion and stability in various solutions, including water, phosphate-buffered saline (PBS), culture medium (CM), and fetal bovine serum (Figure 4H). Meanwhile, TGR-BLDH nanocomposites exhibited excellent stability in PBS with negligible size changes even after 48 h (Figure 4I). Then, the release patterns were evaluated under different conditions, such as in PBS at pH 7.4 (physiological pH), pH 6.0 (close to the pH of the tumor microenvironment and early endosome), and pH 4.5 (similar to the pH of the late endosome/lysosome) (Figure 4J,K). The release process can be generally divided into three stages: (1) an initial rapid release within the first hour, (2) a subsequent sustained slow release over the next few hours, and (3) equilibrium reached after approximately 30 h. The release of the cargo occurs more rapidly at lower pH values. Ultimately, the equilibrium release rate of BLDHs in PBS at pH 4.5 approaches nearly 100%. Moreover, the biocompatibility of BLDHs was studied using a CCK-8 assay. The blank BLDHs did not show any cytotoxicity against LLC cells even when the concentration was as high as 1.5 mg/mL (Figure S4B), which indicated that BLDHs are good candidates for delivery. It was also found that BLDH nanocomposites are biocompatible with blood (Figure S4C). These results proved the excellent stability, biosafety, and bioresponsive capability of BLDH nanocomposites, which is a prerequisite for gene-immune delivery.

### TGR-BLDHs Can Effectively Target, Constrain Cancer Cells, and Stimulate Immune Response

Then, TGR-BLDHs’ targeting efficiency was evaluated. As shown in Figure 5A, the R-BLDHs demonstrated significantly higher cellular uptake efficiency than nonmodified BLDHs. More specifically, the cRGD modification (R-BLDHs) exhibited a more pronounced enhancement in cellular uptake, increasing by 43.3% at 24 h (Figure 5B). Subsequently, TMEM163 expression level (Figure 5C) and CCK8 assays (Figure 5D) were conducted following incubation with various BLDH components. After the integration of DTS, nucleus targeting sequence, and pTMEM163-BLDHs (T-BLDHs) increased TMEM163 transcription levels by 3.5-fold and 2.0-fold for DTS-deficient T-BLDHs (DDTS-T-BLDHs) without or with conjugation of cRGD (DDTS-TR-BLDHs). Moreover, the addition of DTS and cRGD (TR-BLDHs) reduced cell proliferation to 60.6% of that achieved with DDTS-T-BLDHs alone (Figure 5D). Thus, cRGD and DTS synergistically promoted cancer suppression by enhancing the intracellular delivery (Figure 5E).

**Figure 5:**
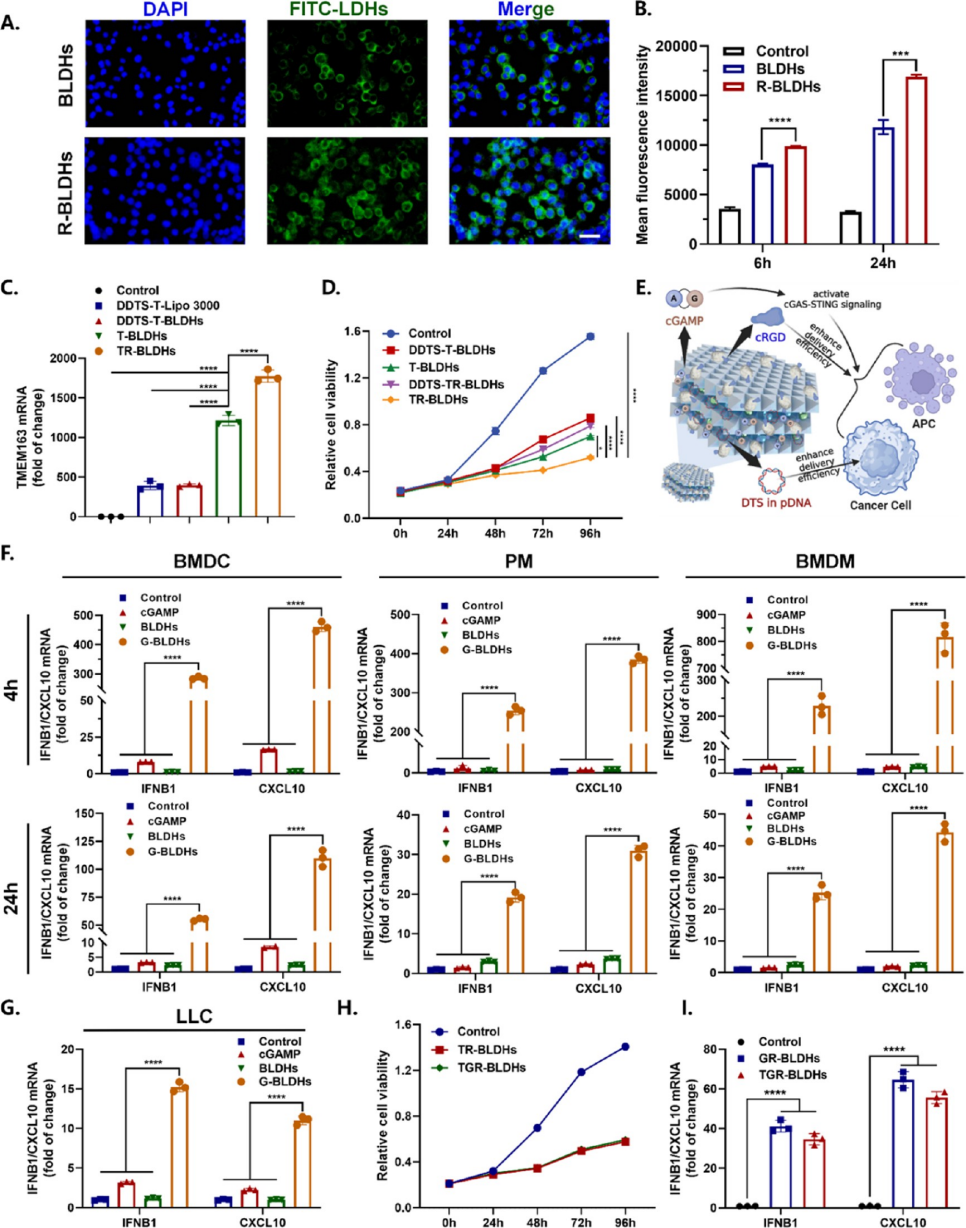
In vitro antitumor properties of TGR-BLDHs. (A) Representative images of cellular uptake efficiency by BSA/LDH nanocomposites with or without cRGD. Scale bar, 50 μm. (B) Cellular uptake efficiency was detected by flow cytometry (*n* = 3). (C) The relative TMEM163 expression of LLC cells treated with different nanocomposites. “DDTS” means deficiency of DTS (*n* = 3). (D) The CCK-8 assays of LLC cells treated with different nanocomposites (*n* = 3). (E) Schematic diagram illustrating the mechanism of action of TGR-BLDHs. (F) The expression of cGAS target genes, including IFNB1 and CXCL10 in BMDC, PM, and BMDM was detected using real-time PCR after 4 h and 24 h of the indicated treatment (*n* = 3). (G) The expression of cGAS target gene of LLC cells treated with different nanocomposites (*n* = 3). (H) The CCK-8 assays of LLC cells treated with different nanocomposites. (I) The expression of cGAS target gene, including IFNB1 and CXCL10 in PM treated with conditioned media culture of LLC cells different nanocomposites (*n* = 3). Statistical significance was determined using a two-tailed Student’s *t*-test. Data are presented as mean ± SEM, *p < 0.05; **p < 0.01; ****p < 0.0001.

Meanwhile, the capability of BLDH-based nanomedicine in evoking antitumor immune response was also investigated in APCs and tumor cells. After incubations with cGAMP-BLDHs (G-BLDHs), IFNB1 and CXCL10 expressions were markedly upregulated in BMDC, PM, and BMDM, while minimal productions were observed in control treatments (blank, soluble cGAMP, or blank-BLDHs) (Figure 5F). Moreover, the G-BLDH treatment significantly induced the secretion of IFN-γ, about 5.0 times that in the control group (Figure S5A). Meanwhile, the interaction between tumor cells and adjacent immune cells also plays a crucial role in activating immunity within the tumor microenvironment. Crosstalk between LLC cells and APCs was investigated by incubating BMDC cells with CM from LLC cells after G-BLDH treatments (Figure S5B). A substantial increase in IFNB1 and CXCL10 mRNA levels was observed in BMDC, similar to direct stimulation, indicating crosstalk between LLC cells and neighboring DCs for STING activation. The expression of cGAS target genes IFNB1 and CXCL10 was also examined in LLC cells directly treated with G-BLDHs (Figure 5G). The results showed that their transcription levels increased (14.7-fold and 10.9-fold, respectively), further corroborating the crosstalk. More importantly, after loading with pTMEM163 and cGAMP, TGR-BLDHs could exhibit distinct antitumor effects and immunostimulatory functions without significantly decreased effectiveness (Figure 5H,I). These findings suggested that TGR-BLDHs could simultaneously achieve effective gene immunotherapy via tumor-targeted delivery of TSG and tumor immune microenvironment remodeling (i.e., STING pathway activation in tumor-infiltrating APCs and all cancerous cells).

### TGR-BLDHs Exhibit Desirable NSCLC Tumor-Targeted Efficacy

Using an in vivo imaging system (IVIS), the biodistribution and tumor-targeting efficiency of TGR-BLDHs were comprehensively investigated. Figure 6A shows real-time tumor images at various time points following intravenous injection of BSA-Cy7, BLDHs^Cy7^, and R-BLDHs^Cy7^. Specifically, the overall fluorescence intensity of R-BLDHs^Cy7^ was significantly higher than that of the other two groups, especially the control group (Figure 6B). The targeting efficiency of the R-BLDHs^Cy7^ group exceeded that of the BSA group by a factor of 2 within the first hour. At 12 h, the accumulation of nanomaterials within the tumor reached its peak, with the accumulation of R-BLDHs^Cy7^ being 2.5 times higher than that of the BSA^Cy7^ group. Moreover, at 24 h postinjection, the fluorescence intensity in the R-BLDHs^Cy7^ group remained high. Mice were sacrificed 24 h after injection, and the fluorescence intensity of excised organs (heart, liver, spleen, lungs, kidneys, and tumor) was meticulously examined. Regarding biodistribution, the results indicated that R-BLDHs^Cy7^ could effectively accumulate inside tumors (Figure 6C,D). Notably, about 10.8% of R-BLDHs^Cy7^ accumulated in the tumor at 24 h postinjection, significantly higher than in other organs (heart, liver, spleen, lungs, or kidneys) (Figure 6E). These findings suggest that introducing cRGD facilitates the accumulation of BLDHs in tumors, consistent with cellular uptake results in vitro.

**Figure 6:**
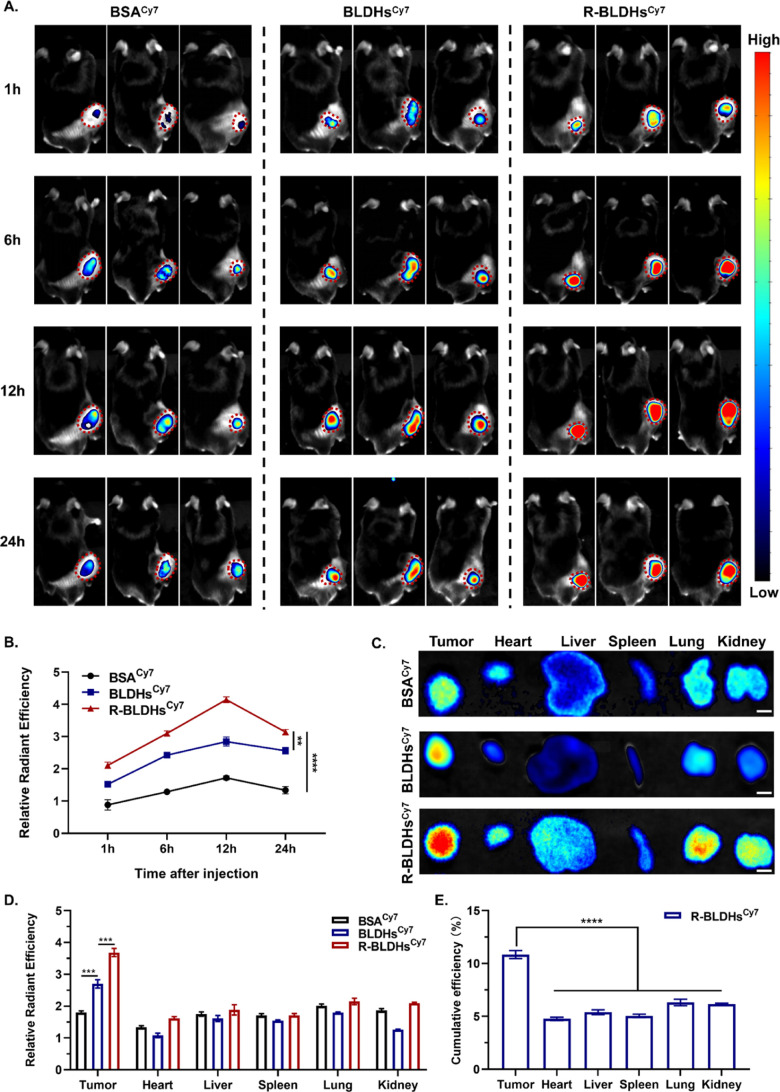
In vivo cancer-targeting ability of TGR-BLDHs (*n* = 3). (A) Real-time imaging of in vivo tumor targeting in each group of tumor-bearing mice at different times (*n* = 3). (B) Relative radiant efficiency of tumors in each group of mice at different time points. Relative radiant efficiency refers to the ratio of the measured fluorescence intensity of each group at each time point to that of the BSA^Cy7^ control group at 1 h, which was used as the baseline (set to 1.0). (C) Fluorescence images of tumors and major organs of mice in each group 24 h after injection. Scale bar, 5 mm. (D) Relative radiant efficiency of tumors and major organs of mice in each group 24 h after injection. (E) Cumulative efficiency of tumors and major organs compared with the original injected nanomaterials. Statistical significance was determined using a two-tailed Student’s *t*-test. Data are presented as mean ± SEM, *p < 0.05; **p < 0.01; ****p < 0.0001.

### TGR-BLDHs Successfully Generate Potent Gene Immunotherapy against NSCLC Tumor In Vivo

The in vivo antitumor efficacy of the TGR-BLDHs was evaluated on a subcutaneous cancer xenograft model (Figure 7A). As shown in Figure 7B,C, TR-BLDHs generated a moderate antitumor effect with a 62.6% decrease in tumor volumes, which was better than GR-BLDHs. pTMEM163/cGAMP hardly inhibited tumor growth, the tumor volume of which was similar to that of the PBS-treated group (Figure S6A). Importantly, TGR-BLDHs exhibited extraordinary antitumor activity (84.0% decrease). The enhanced antitumor activity might be attributed to the activated cGAMP/STING signal. At 14 days postinjection, sizes of tumors after different treatments intuitively confirmed the excellent antitumor activity of the nanocomposites (Figure 7D). The average tumor weights were 1.5, 0.6, 0.9, and 0.2 g for the mice treated with different formulations, respectively (Figure 7E). In addition, the body weight of the mice remained similar after various treatments of the BLDH-based formulation, indicating relatively low side effects (Figure 7F).

**Figure 7:**
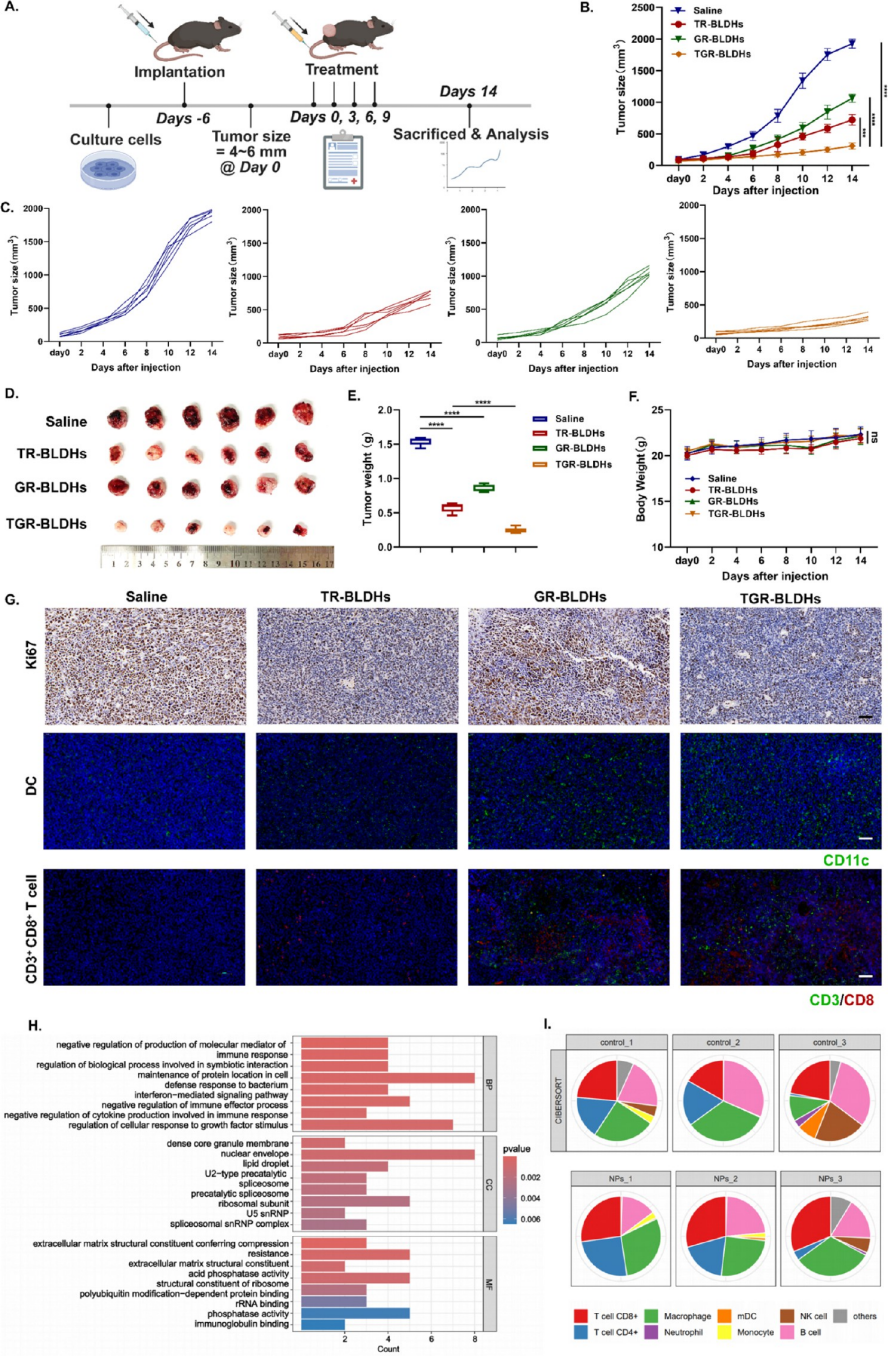
The nanocomposite enhances the antitumor effect in xenograft tumor model. (A) Schematic illustration of the administration design. (B) Photograph of the tumor extracted from mice after the indicated treatments. (C–E) The detection of tumor volume (C), tumor weight (D), and body weight (E) after different treatments (*n* = 6). (F) The detection of tumor size of each mouse in different treatment groups. (G) HE staining and immune cells immunofluorescence of tumors extracted from mice after the indicated treatments. Scale bar, 50 μm. (H) GO functional enrichment analysis. (I) Proportion of immune cells for each sample. Statistical significance was determined using a two-tailed Student’s *t*-test. Data are presented as mean ± SEM, *p < 0.05; **p < 0.01; ****p < 0.0001.

Meanwhile, it was found that the proportion of DC, CD8^+^ T cells increased in tumor tissues with the effect of cGAMP as determined by immunofluorescence (IF) (Figure 7G). Subsequently, *K*_i_-67 lHC staining was carried out. The results were in accordance with the tumor growth inhibition curves. Moreover, proteomic sequencing was performed on mouse tumor samples, revealing the enrichment of several immune-related pathways (Figures 7H and S7A). The comprehensive nature of proteomics allows for an in-depth understanding of protein expression and modifications, providing insights into the functional state of cells and tissues. This analysis indicated a notable increase in CD8+ T cells and macrophages following TGR-BLDH treatment, suggesting enhanced immune activation and antitumor effects (Figure 7I).

### TGR-BLDHs Further Suppress Tumor Progression in a Lung-Metastasis Model

To further validate the therapeutic efficacy of TGR-BLDHs in a physiologically relevant setting, we established an LLC lung metastasis model that mimics the pulmonary microenvironment of NSCLC (Figure 8A). Mice bearing lung metastases received saline, TR-BLDHs, GR-BLDHs, or TGR-BLDHs via systemic administration every 3 days four times.

**Figure 8:**
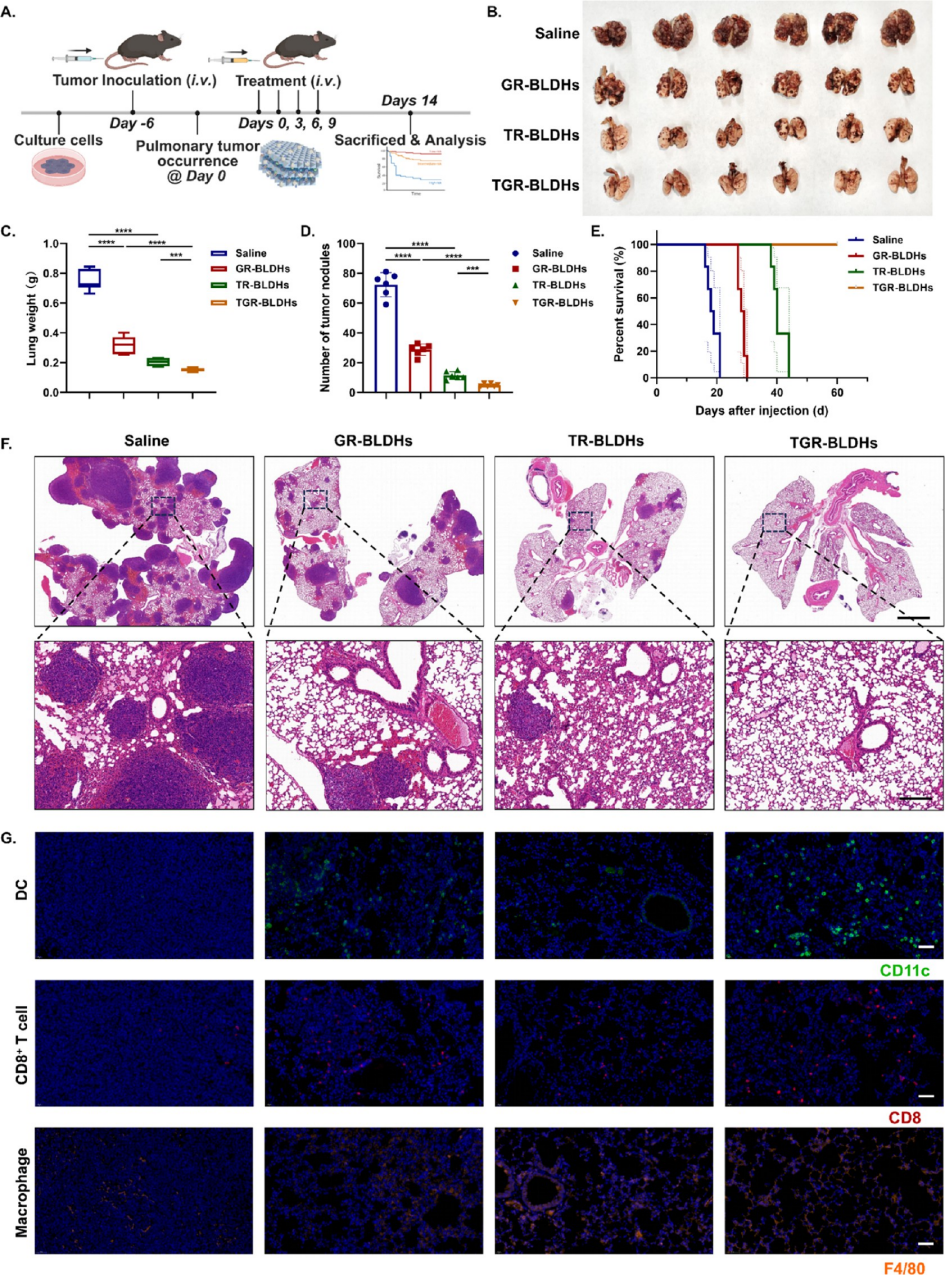
TGR-BLDHs effectively inhibit growth of pulmonary tumor and stimulate antitumor immunity in a lung-metastasis LLC model. (A) Schematic illustration of the experimental schedule for the lung-metastasis model treatment. (B) Representative photographs of excised lungs from mice after 14 days of treatment with saline, GR-BLDHs, TR-BLDHs, or TGR-BLDHs. (C) Average lung weights of each group on day 14 (*n* = 6). (D) Quantitative analysis of metastatic nodules per lung after different treatments (*n* = 6). (E) Kaplan–Meier survival curves of mice treated with different formulations (*n* = 6). (F) Representative H&E-stained sections of lung tissues showing tumor morphology and lesion distribution. Scale bar, 2000 μm. Enlarged scale bar, 200 μm. (G) Immunofluorescence staining of lung tissues showing infiltrated DCs (CD11c^+^), cytotoxic T cells (CD8^+^), and macrophages (F4/80^+^) in each group. Scale bar, 50 μm. Statistical significance was determined using a two-tailed Student’s *t*-test. Data are presented as mean ± SEM, *p < 0.05; **p < 0.01; ****p < 0.0001.

Macroscopic evaluation revealed pronounced differences among the four groups (Figure 8B). Lungs from the saline-treated group were densely covered with metastatic nodules, while TR-BLDHs and GR-BLDHs significantly reduced the tumor burden. Remarkably, mice treated with TGR-BLDHs displayed almost complete inhibition of pulmonary metastases, with lungs appearing macroscopically normal. Consistently, lung weights at day 14 (Figure 8C) decreased progressively from saline (0.75 g) to TR-BLDHs (0.32 g), GR-BLDHs (0.20 g), and TGR-BLDHs (0.15 g), confirming the superior therapeutic efficacy of our dual-functional nanoplatform. Quantitative analysis of metastatic nodules per lung (Figure 8D) also demonstrated a statistically significant decrease in the number of lesions, with TGR-BLDHs reducing visible nodules by above 93% compared to saline.

In the same parallel experiment, survival analysis further supported these findings (Figure 8E). Mice treated with TGR-BLDHs exhibited the longest survival, exceeding 60 days, whereas the other groups showed markedly shorter lifespans, demonstrating that combined tumor-suppressor gene restoration and STING activation achieved a durable therapeutic benefit.

Histological and immunofluorescence analyses provided further mechanistic insight. H&E staining confirmed that TGR-BLDH-treated lungs contained only scattered residual tumor foci with preserved alveolar structures, while other groups exhibited extensive tumor infiltration (Figure 8F). Immunofluorescence staining revealed significantly elevated infiltration of DCs, cytotoxic CD8^+^ T cells, and macrophages in the TGR-BLDH group, consistent with an enhanced antigen presentation and STING-mediated immune activation (Figure 8G).

Collectively, these results demonstrate that TGR-BLDHs not only effectively inhibit pulmonary metastasis but also stimulate robust systemic antitumor immunity, confirming their translational potential for NSCLC therapy.

### The Excellent Biocompatibility of TGR-BLDHs

Finally, the biosafety of various BLDH nanocomposites was rigorously evaluated. Fourteen days after intravenous injection, major organs, including the heart, liver, spleen, lungs, and kidneys, were harvested and subjected to hematoxylin and eosin (H&E) staining. The histopathological examination (Figure 9A) revealed no significant abnormalities or toxic effects in any of these organs, indicating that the TGR-BLDH nanocomposites did not induce histopathological toxicity.

**Figure 9:**
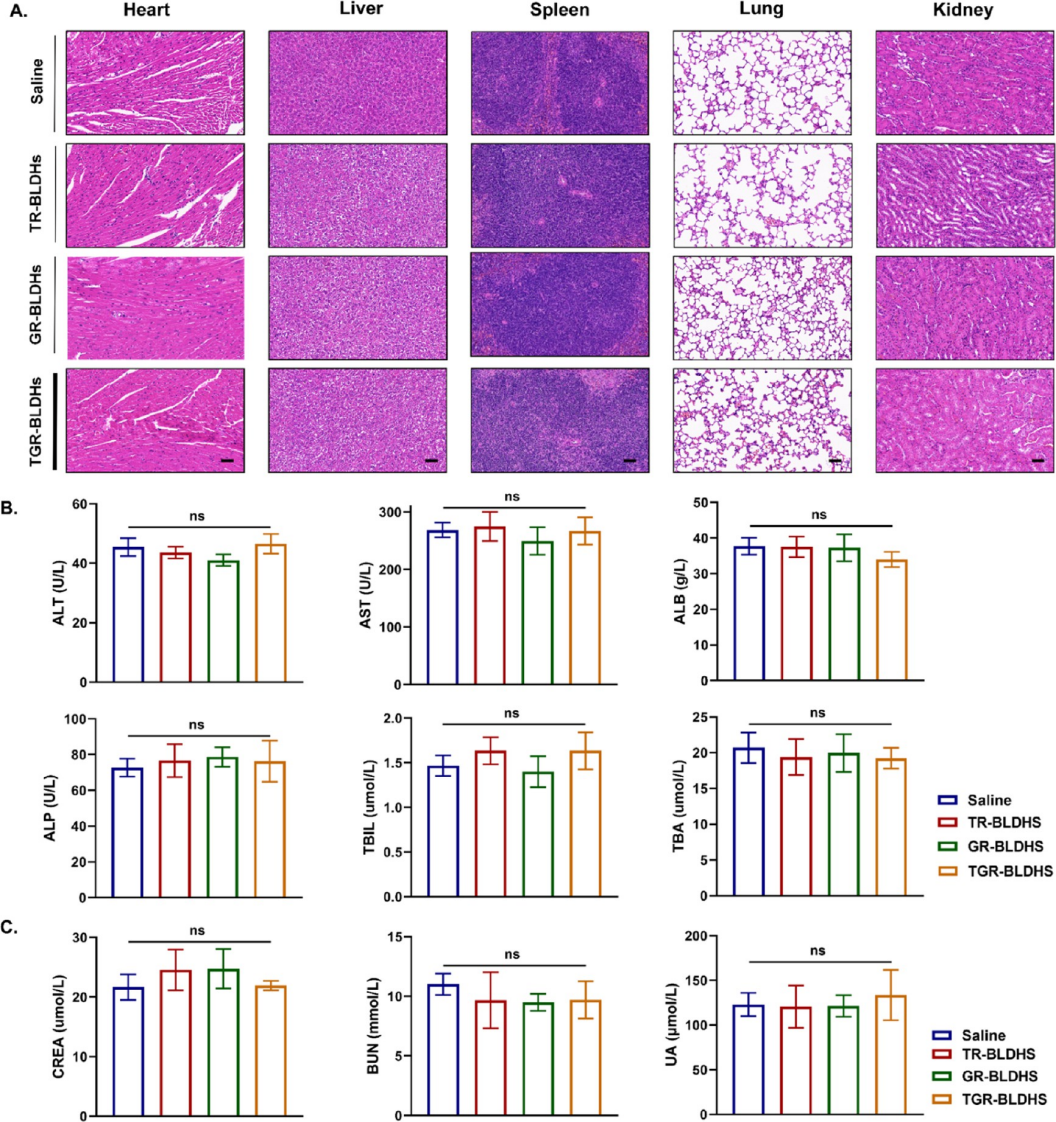
Optimal histopathological and biochemical safety profile of TGR-BLDHs in vivo (*n* = 6). (A) H&E staining of vital organs 14 days after different treatments. Scale bar, 50 μm. (B,C) Serological biochemical analysis including liver (B) and kidney (C) function test in LLC tumor model after the treatment. ALT, alanine aminotransferase; AST, aspartate aminotransferase; ALB, albumin; ALP, alkaline phosphatase; TBIL, total bilirubin; TBA, total biliary acid; CREA, creatinine; BUN, blood urea nitrogen; UA, uric acid. Statistical significance was determined using a two-tailed Student’s *t*-test. Data are presented as mean ± SEM, *p < 0.05; **p < 0.01; ****p < 0.0001.

Furthermore, a thorough serological biochemical analysis was carried out to evaluate the potential effects of the nanocomposites on liver and kidney functions. The liver function assessments comprised measurements of alanine aminotransferase (ALT), aspartate aminotransferase (AST), albumin (ALB), alkaline phosphatase (ALP), total bilirubin (TBIL), and total biliary acid (TBA). Similarly, kidney function was assessed by determining the levels of creatinine (CREA), blood urea nitrogen (BUN), and uric acid (UA). The results indicated that all liver function parameters (Figure 9B) and renal function markers (Figure 9C) fell within the normal physiological range, suggesting that the TGR-BLDHs did not induce liver damage or dysfunction or impair kidney function. These collective findings demonstrate the excellent biocompatibility and low systemic toxicity of TGR-BLDH nanocomposites.

## Discussion

Despite recent advances in cancer therapy, lung cancer persists as a formidable challenge in oncology, with current treatments often being limited by insufficient targeting specificity and suboptimal efficacy. To address these challenges, we developed TGR-BLDHs, a novel gene immunotherapy strategy for NSCLC. This innovative targeted nanomedicine combined a newly confirmed NSCLC tumor suppressor gene (TMEM163) with the STING agonist (cGAMP), delivered via cyclic RGD-modified bovine serum albumin/layered double hydroxides (cRGD-BSA/LDHs). TGR-BLDHs directly impeded tumor growth via TMEM163-mediated pathways while simultaneously reshaping the tumor immune landscape through STING pathway activation, showcasing superior tumor volume reduction and immune cell infiltration compared to monotherapies. As-prepared TGR-BLDHs offer a promising strategy for overcoming multiple challenges inherent in existing cancer treatments via the integration of desirable targeting efficiency, tumor-specific inhibition, and comprehensive immune stimulation.

Gene therapy and immunotherapy have emerged as promising alternatives to traditional cancer treatments, but their effectiveness is generally constrained as a sole therapeutic approach. For instance, a clinical trial utilizing intratumoral adenoviral p53 gene therapy in advanced NSCLC patients undergoing first-line chemotherapy hardly offers additional benefit, showing the objective response rates for lesions at 52% and 48% with or without p53 treatment, respectively.^27^ Similarly, single-agent immunotherapies, such as PD-1 inhibitors, only demonstrated 14–20% long-lasting disease response rates in NSCLC patients.^28^ Notably, a preclinical study combined p53 gene therapy with PD-1 blockade in a primary cancer mouse model, demonstrating enhanced tumor regression and increased survival compared to either monotherapy.^29^ These findings highlight gene immunotherapy’s great potential in treating cancer with improved efficiency.

In our study, TMEM163, a zinc-binding protein, was first identified as a potent TSG for NSCLC, exhibiting desirable efficacy, even compared with several well-known TSGs (Figures 1 and 3). For example, p53 gene therapy inhibited lung cancer cell growth by 60% in vitro,^30^ while the overexpression of TMEM163 reduced cell growth rates to 57.9% and 56.3% in A549 and H1975 cells, respectively (Figure 2B). Moreover, overexpressing TMEM163 demonstrated a remarkable ability to suppress tumor growth in vivo with negligible growth by the third week (Figure 2I,J). Such tumor-suppressed effectiveness was comparable to or even surpassed that observed with other TSGs like MTSS1 or PTEN gene, which has been reported to reduce tumor volume by approximately 65% or 60% in NSCLC xenograft models, respectively.^31,32^ Interestingly, TMEM163 positively correlates with STING1 expression (Figure S3B), suggesting the potential to induce a synergistic effect by involving immunotherapy via the STING pathway activation.

To achieve a combinatorial treatment strategy, a novel nanomedicine, TGR-BLDHs was developed for targeted restoring TMEM163 function and stimulating antitumor immune responses simultaneously. As-prepared nanosystem was able to achieve efficient coloading of both pTMEM163 and cGAMP and also facilitate endosomal escape, a critical step in intracellular drug delivery. The pH-dependent dissolution of LDHs in the acidic environment of endosomes led to several beneficial effects: (1) it triggered the release of the therapeutic cargo, (2) the dissolution products (Mg^2+^ and Al^3+^ ions) increased the osmotic pressure within the endosome, promoting its rupture,^33,34^ and (3) the hydroxide ions generated neutralized the endosomal pH, protecting the cargo from degradation. This proton sponge effect significantly enhances the cytoplasmic delivery of psTMEM163 and cGAMP, maximizing their therapeutic efficacy.^35^ The pH-dependent release profile of our nanoplatform (Figure 4J,K) ensured minimal cargo leakage during circulation (pH 7.4) while enabling rapid release during the endosome pathway (pH 4.0–6.0 in lysosome or endosome) after internalization. This controlled release behavior was crucial for maximizing the therapeutic effect while minimizing systemic toxicity.

Meanwhile, the TGR-BLDHs demonstrated effective tumor targeting and cellular uptake, with 43.3% increased cellular uptake at 24 h (Figure 5A,B), comparable to or slightly better than many other reported cRGD-modified nanoparticles. For instance, a cRGD-modified liposome for siRNA delivery in LLC cells increased cellular uptake by 40%.^36^ The in vivo biodistribution results further highlighted the superior targeting efficiency of our nanoplatform. R-BLDHs^Cy7^ demonstrated 2.5 times higher tumor accumulation compared to the control group at 12 h postinjection, with 7.7% ID/g (injected dose per gram) still present in the tumor at 24 h (Figure 6C,E). This level of tumor accumulation was notably higher than that reported for other nanoplatforms, such as cRGD-conjugated polymers, which showed tumor accumulation of roughly 2% ID/g within 24 h.^37^

In addition, TRG-BLDHs also showed superior efficiency compared to other nanocarriers used for cargo delivery.^18^ Previously, cGAMP-loading phosphatidylserine-coated liposomes mildly increased IFNB1 and CXCL10 mRNA expression compared to free cGAMP, with a few-fold change.^38^ In contrast, TGR-BLDHs induced robust upregulation of IFNB1 and CXCL10 by several 100-fold in various immune cells. More importantly, TRG-BLDHs were able to drive cancerous cells (LLC cells), the largest cell populations in malignant tissues, to secrete antitumor cytokines with increased expression levels by 14.7-fold and 10.9-fold, respectively (Figure 5F,G). Meanwhile, TRG-BLDHs also enhanced TMEM163 expression and mediated a concomitant increase in antiproliferative effects (Figure 5C,D). Therefore, after tumor-targeted accumulation, TRG-BLDHs were able to evoke potential antitumor effects via TSG-mediated suppression and antitumor immunity (immune microenvironment remodeling).

The efficacy of our approach was evident in the in vivo xenograft model results (Figure 7). TGR-BLDHs treatment led to an 84.0% decrease in tumor volume, significantly outperforming single-agent therapies. The immunostimulatory effects of TGR-BLDHs extended beyond activation of the STING pathway in cancer cells. Immunofluorescence analysis of tumor tissues revealed increased infiltration of DCs and CD8+ T cells following TGR-BLDH treatment (Figure 7G). This enhanced immune cell infiltration is crucial in converting “cold” tumors into “hot” tumors, thereby enhancing the overall antitumor immune response. The proteomic analysis of tumor samples further supported the immunomodulatory effects of the TGR-BLDHs. The enrichment of immune-related pathways and the increase in CD8+ T cells and macrophages following treatment (Figure 7H,I) indicated a comprehensive remodeling of the TIME. This multifaceted immune activation distinguished our approach from many other gene therapy strategies that primarily focus on direct tumor cell killing. The observed immune activation could be attributed to several factors. First, the TMEM163-mediated tumor suppression likely induced immunogenic cell death, releasing tumor-associated antigens that can prime the immune system. Second, the cGAMP payload activated the STING pathway in both tumor and tumor-infiltrating immune cells, particularly APCs. This dual activation of innate and adaptive immune responses created a robust antitumor immune environment. Notably, our system may also benefit from the inevitable clearance of some nanoparticles by peripheral immune cells. While this clearance was often seen as a limitation for nanoparticle-based therapies, in our case, it may contribute to systemic immune activation. Nanoparticles cleared by peripheral APCs can still deliver their cGAMP payload, activating these cells and potentially inducing a systemic antitumor immune response. This inevitable “off-target” effect may enhance the overall therapeutic efficacy by priming the immune system beyond the local tumor environment. Given that TMEM163 is a TSG, potential off-target effects, if any, are unlikely to exert toxic effects on various body organs (Figure 9). Furthermore, the therapeutic and immunomodulatory efficacy of TGR-BLDHs was further corroborated in the lung-metastasis LLC model (Figure 8). In this more physiologically relevant setting, TGR-BLDHs treatment markedly decreased the tumor burden by over 90% relative to the control, prolonged survival, and promoted extensive infiltration of DCs and CD8^+^ T cells within metastatic foci. These results demonstrated that our nanoplatform maintains therapeutic efficacy within the complex pulmonary microenvironment, where both vascular and immune barriers often hinder drug delivery. The consistent results between subcutaneous and lung-metastasis models not only confirmed the systemic robustness of our dual-functional nanoplatform but also highlighted its potential for treating disseminated or metastatic cancer, reinforcing the translational value of this approach for NSCLC management.

In the clinic, several delivery agents have been used for gene therapy, ranging from adeno-associated virus (AAV) capsids to nonviral vectors (e.g., liposomes).^39^ However, therapeutic outcomes are generally unsatisfactory. For instance, a study using AAV capsid for codelivery of a TSG (PTEN) and an immunostimulatory CpG oligonucleotide exhibited minimal tumor inhibition, showing no significant difference from the control group.^40^ Moreover, many gene therapy vectors, particularly viral vectors, can induce significant immune responses and toxicity. It is notable that TGR-BLDH nanomedicine was able to achieve an 84% reduction in tumor volume (Figure 7B,D), with desirable biocompatibility (Figure 9). This favorable therapeutic efficiency and safety profile, especially the albumin-based synthesized strategy, positions the TGR-BLDHs as promising clinical translation candidates. Consequently, the TGR-BLDH nanomedicine represents a significant advancement in cancer gene immunotherapy.

## Conclusion

In conclusion, our work first illustrates and emphasizes the anticarcinogenic role of TMEM163 in NSCLC tumorigenesis. Based on this, a novel nanomedicine, TGR-BLDHs was designed for precision gene immunotherapy. As-prepared TGR-BLDHs simultaneously restored the TMEM163 function and remodeled the TIME, thereby effectively suppressing tumor growth. This study introduces an innovative strategy to enhance gene immunotherapy, establishing a potent and adaptable nanomedicine designed for precise lung cancer therapy.

## Methods and Experimental

### NSCLC Tissues

A total of seventy-five specimens of NSCLC tissues, along with corresponding tumor-adjacent normal lung tissue samples, were collected from the Second Affiliated Hospital, College of Medicine, Zhejiang University (Hangzhou, China). These specimens were histologically confirmed. Of these, 35 fresh tissue specimens were utilized for mRNA expression analysis of TMEM163, while the remaining 40 paraffin-embedded tissues were subjected to immunohistochemical staining. This research was conducted under the approval of the Research Ethics Committee of the Second Affiliated Hospital, College of Medicine, Zhejiang University (Hangzhou, China, No. 2023–0259), and adhered to the principles of the Declaration of Helsinki. Informed written consent was acquired from all participants prior to their inclusion in the study.

### Animals and Animal Ethics

All animal experiments were approved by the Animal Care and Use Committee of the Zhejiang University School of Medicine (Approval No. ZJU20230010). C57BL/6J mice were procured from Charles River Laboratories (Zhejiang, China) to ensure a consistent genetic background. Male mice of 6–8 weeks were selected for the experiments to maintain age and gender matching. Experimental procedures adhered to the guidelines provided by the National Institutes of Health for the care and use of laboratory animals (NIH Publications No. 8023, revised 1978) and were approved by the Institute of Process Engineering, Chinese Academy of Sciences and the Animal Care and Use Committee of the Zhejiang University School of Medicine. Sample sizes for experimental groups were determined based on statistical power, feasibility, and ethical considerations. Techniques and procedures were optimized to prioritize animal comfort and minimize stress.

### Synthesis of BSA/LDH-Based Nanocomposites

Mg_2_Al-LDH nanoparticles and BLDHs were synthesized according to previously established protocols. LDHs with an average size of 110 nm were prepared and dispersed in deionized water. The LDHs suspension was subjected to heat treatment in an autoclave at 100 °C for 16 h. LDHs were gradually added to a BSA solution (mass ratio of BSA/LDHs = 5:2), stirred for 30 min, and centrifuged at 20,000*g* for 20 min. The pellet was resuspended and further processed by the addition of pDNA and cGAMP.

### In Vivo Antitumor Therapy in Xenograft Model and Analysis

For the antitumor study, a subcutaneous lung cancer model was established using LLC cells (1 × 10^6^) mixed with Matrigel and injected into the abdominal region of C57BL/6J mice. Once tumors reached an approximate volume of 50 mm^3^, mice were randomly assigned to treatment groups. Nanomaterial solutions were administered intravenously on days 0, 3, 6, and 9. Tumor volume and body weight were measured every 2 days, with tumor volume calculated using the formula: *D* × *d* ^2^/2 (mm^3^), where *D* is the longest diameter and d is the shortest diameter. On day 14, tumors were excised, photographed, weighed, and subjected to histological analysis via hematoxylin and eosin (H&E) staining and Ki67 immunostaining to assess proliferation and apoptosis.

## Supplementary Material



## Data Availability

All the data necessary to support the findings and conclusions presented in this paper are included within the main text and the Supporting Information. The original source data are available in this paper.

## Notes

Additional experimental sections; cell lines; cell transfection and generation of engineered cell lines; cell proliferation assay; quantitative RT-PCR; RNA-sequencing and data analysis; Western blotting; synthesis and characterization of BSA/LDH-based nanocomposites; cell targeting assays; assessment of in vitro cytotoxicity; evaluation of in vitro immune activation; in vivo imaging of TGR-BLDHs; multiple immunofluorescence staining; tumor proteomics analysis; in vivo lung metastasis tumor model; biocompatibility; and statistical analysis (PDF)
