# Supplementary material for: Synergistic Gene Immunotherapy for Lung Cancer via Targeted Nanomedicine Restoring Genetic Tumor Suppression and Activating STING Pathway
Source: ACS Nano. 2026 Feb 12;20(7):6138–55. doi: 10.1021/acsnano.5c20264 (PMC12947727; doi:10.1021/acsnano.5c20264)
Supplement: Supplementary file 1 [file nn5c20264_si_001.pdf]

Supporting information

Synergistic Gene Immunotherapy for Lung Cancer via Targeted Nanomedicine Restoring Genetic Tumor Suppression and Activating STING Pathway

Weiyu Chen[1,2 #], Xinjie Zheng[1#], Yuan Wu[1,3], Yiming Xu[1], Hangqi Ni[1], Peng Xiao[4], Weibo Cai[5*], Kai Wang[1,2*]

[1] Department of Respiratory and Critical Care Medicine, Center for Oncology Medicine, the Fourth Affiliated Hospital of School of Medicine, and International School of Medicine, International Institutes of Medicine, Zhejiang University, Yiwu, China, 322000.

[2] Zhejiang Key Laboratory of Precision Diagnosis and Treatment for Lung Cancer, Yiwu, China, 322000.

[3] College of Jiyang, Zhejiang A&F University, Zhuji, 311800, China

[4] Department of Gastroenterology, Sir Run Run Shaw Hospital, Zhejiang University School of Medicine, Hangzhou 310016, China.

[5] Departments of Radiology and Medical Physics, University of Wisconsin-Madison, Madison, Wisconsin, 53705, United States

"#" These authors contributed equally to this work.

Correspondence:

Prof. Kai Wang

Email: kaiw@zju.edu.cn

Prof. Weibo Cai

E-mail: wcai@uwhealth.org

Table of Contents

1. Additional Experimental Sections

Materials and methods

2. Supporting Figures

Figure S1. TMEM163 does not affect the cell cycle and apoptosis of lung cancer cells. A) Cell cycle assays of A549 cells with TMEM163 overexpression. B) Apoptosis assays of A549 cells with TMEM163 overexpression. Two-tailed student's t test was performed for statistical significance. Error bars represent mean ± SEM, ns, not significant.

Figure S2. Volcano plot and heat map of differentially expressed genes.

Figure S3. Prediction analysis of TMEM163 using relevant datasets. A) The strong interaction forces between MAPK14 and TMEM163. B) Correlation between TMEM163 TPM and STING1 TPM.

Figure S4. The high loading efficiency and biocompatibility of BSA/LDHs has been proven to be excellent. A) The assay of loading content of pDNA and cGAMP in BSA/LDHs. B) CCK-8 assay of BSA/LDHs with different concentration. C) Hemolysis test of BSA/LDHs with different concentration.

Figure S5. G-BLDHs nanomaterials promote the cGAS/STING signaling transduction. A) The cytokine concentration in the culture medium of BMDM and BMDC cells with the indicated treatment was detected using an ELISA kit after 12 h of the indicated treatment. B) The expression of IFNB1 and CXCL10 in BMDC cells incubated with CM from LLC cells after G-BLDHs treatments was detected using real-time PCR after 12 h of the indicated treatment.

Figure S6. Therapeutic effect of free pTMEM163 and cGAMP on xenograft tumor models.

Figure S7. Volcano plot and clustering heat map of differential protein. Red indicates significantly up-regulated proteins, blue indicates significantly down-regulated proteins.

3. Supporting Tables

Table S1. List of RT-PCR primer sequences

Table S2. Antibody

1. Additional Experimental Sections

Cell Lines

A549, H1975, and Lewis Lung cancer (LLC) cell lines were sourced from the China Infrastructure of Cell Line Resources (Beijing, China). A549 and H1975 cells were cultured in RPMI-1640 medium (Gibco Invitrogen) with 10% fetal bovine serum (FBS; Invitrogen) and 100 U/mL penicillin/streptomycin (Invitrogen). Cells were incubated in a 5% CO2 humidified incubator at 37 °C. Similarly, LLC cells were maintained in Dulbecco's Modified Eagle Medium (DMEM) (Gibco Invitrogen) under identical conditions, supplemented with 10% FBS and 100 U/mL penicillin/streptomycin. All the cell lines were free of mycoplasma contamination.

Cell Transfection and Generation of Engineered Cell Lines

Cell Transfection: Lipofectamine 3000 (Invitrogen, L3000015) was used to transfect cells with plasmids following the manufacturer's protocol. The culture medium was refreshed 4-6 hours post-transfection, and transfected cells were utilized for further assays after 24 to 48 hours.

Generation of engineered cell lines: To generate engineered cell lines, HEK293T cells were co-transfected with the HBLV-TMEM163-ZsGreen-PURO plasmid using a transfection reagent (Hanbio Biotechnology). The culture medium was replaced 6-8 hours after transfection. After 48 hours, the supernatant was harvested, filtered through a 0.45-μm membrane, and stored at -80°C until use. Cells were infected with the recombinant virus in the presence of 8 μg/ml polybrene (Sigma) and selected with 1 μg/ml puromycin (Sigma) for one week to ensure stable integration.

Cell Proliferation Assay

For the CCK-8 assay, 2,000 cells per well were plated in 96-well plates and treated accordingly the following day. The CCK-8 reagent (Beyotime, C0039)

was added at designated time points (0, 24, 48, 72, and 96 hours post-treatment), and absorbance at 450 nm was recorded to evaluate cell viability and proliferation. The EdU assay was conducted using the BeyoClick™ EdU-488 kit (Beyotime, C0071S), with cells seeded at a density of 1.5 × 10^5 cells/well in 6-well plates one day before treatment. Colony formation assays involved seeding 1,000 cells in 6-well plates and incubating them under the specified treatment conditions for 14 days. Colonies exceeding 200 μm in diameter were stained with crystal violet and counted. Migration assays were performed by plating 2 × 10^4 cells into Boyden chambers (8-μm pore, Corning), with migration assessed after 24 hours by staining the migrated cells with crystal violet. For wound healing assays, cells were plated at 3 × 10^5 cells/well, and linear wounds were created post-serum starvation. Wound closure was monitored over 72 hours, with images captured at 0, 24, and 72 hours.

Quantitative RT-PCR

Total RNA was extracted from cells using the RNA extraction reagent (Vazyme, RC101-01) according to the manufacturer's protocol. cDNA was synthesized from 1 μg of RNA using the HiScript quantitative RT-PCR kit (Vazyme, R333-01). Quantitative PCR was performed using SYBR Premix Ex Taq™ II (Takara, RR820A) on a LightCycler Roche480 instrument (Roche). Gene expression levels were normalized to β-actin, and relative expression was determined using the $2^{-\Delta\Delta Ct}$ method. Primer sequences are provided in Table S1.

RNA-Sequencing and Data Analysis

A549 cells were transfected with a TMEM163-encoding plasmid or empty vector control using Lipofectamine 3000 (Invitrogen). Overexpression was verified by qRT-PCR and Western blot analysis 48 hours post-transfection. Total RNA was extracted using TRIzol reagent (Invitrogen), and samples with RIN > 8 were used for library preparation. Paired-end sequencing (150 bp) was performed on an Illumina NovaSeq 6000 platform. Differential gene expression

analysis was conducted using DESeq2 (adjusted p-value < 0.05, |log2FoldChange| > 1). Gene Ontology (GO), Kyoto Encyclopedia of Genes and Genomes (KEGG) pathway enrichment, and Gene Set Enrichment Analysis (GSEA) were performed to identify significantly enriched gene sets and pathways.

Western Blotting

Proteins were extracted from cells and tissues using RIPA lysis buffer (Beyotime), and protein concentration was quantified via BCA assay (Beyotime). After boiling at 100°C, samples were resolved on 12.5% SDS-PAGE gels and transferred to nitrocellulose membranes. Membranes were incubated with primary antibodies overnight at 4°C and then washed and incubated with secondary antibodies at room temperature. The list of primary antibodies used can be found in Table S2. Protein bands were visualized with the Odyssey system.

Synthesis and Characterization of BSA/LDHs-based Nanocomposites

Plasmid DNA (pTMEM163) was first adsorbed into BLDHs nanoparticles at a feeding mass ratio of 40:1 (carrier:pDNA). The suspension was stirred gently at room temperature to allow anion exchange and electrostatic binding. After centrifugation (20,000 g, 20 min), the pDNA-loaded BLDHs were redispersed in solution containing cGAMP at a mass ratio of 1:1 (relative to pDNA). The mixture was incubated for under gentle stirring to allow surface adsorption through hydrogen bonding and protein interaction. The final product (TGR-BLDHs) was collected by centrifugation and redispersed for subsequent experiments.

The morphology of the nanoparticles was examined by transmission electron microscopy (TEM; HT7700, 80 kV). Dynamic light scattering (DLS; Malvern Instruments) was used to analyze the size distribution. The loading capacity of pDNA and cGAMP in BLDHs nanoparticles was determined by adding BLDHs

to solutions with varying solute concentrations, stirring for 20 minutes, centrifuging at 20,000 g for 20 minutes, and quantifying the residual concentrations in the supernatant.

Cell Targeting Assays

The addition method of DTS was to separately add the 3NF1 sequence and 3NF2 sequence before and after TMEM163 in the plasmid: 3NF1 : 5'-CTGGGGACTTTCCAGCCTGGGGACTTTCCAGCTGGGACTTTCCAGG-3'; 3NF2 : 5'-CTGGGGACTTTCCAGCTGGGGACTTTCCAGCTGGGACTTTCCAGGAG-3'. 3NFs, which are abbreviated nucleotide sequences, have been engineered to effectively bind to NF-κB, a crucial transcription factor. These optimized DNA motifs facilitate improved nuclear translocation of plasmids that incorporate them in their structure.

Target cells were seeded in 12-well plates at a density of 1×10^6 cells per well and treated with FITC-labeled nanomaterials. Cellular uptake and localization were analyzed using confocal microscopy, while flow cytometry was used to quantify the mean fluorescence intensity. Statistical analyses were performed to compare the targeting efficacy between control and treated groups.

Assessment of In vitro Cytotoxicity

The potential cytotoxic effects of BLDHs were evaluated using the cell counting kit-8 (CCK-8) assay. Cells were seeded in culture media at a concentration of 3×10^4 cells per milliliter and allowed to adhere for 24 hours. Various concentrations of BLDHs were added to the wells, and the cells were incubated for an additional 48 hours. Following three washes with PBS, 10 μL of CCK-8 reagent was introduced into each well, and the cells were incubated for 2 hours. The absorbance at 450 nm was measured using a microplate reader to determine cell viability.

Evaluation of In vitro Immune Activation

Bone marrow-derived macrophages (BMDM), bone marrow-derived dendritic cells (BMDC), and peritoneal macrophages (PM) were prepared as previously described. The cells were seeded in 12-well plates at a density of 1 × 10^6 cells per well, after which G-BLDH nanocomposites were introduced and incubated for either 4 or 24 hours. Gene expression was assessed using quantitative RT-PCR, while supernatants were collected for cytokine analysis, with interferon-gamma (IFN-γ) detected using ELISA. mRNA levels of IFNB1 and CXCL10 were also measured to evaluate immune response.

In vivo Imaging of TGR-BLDHs

Subcutaneous LLC tumor-bearing mice were administered BSA^{Cy7}, $BLDHs^{Cy7}$, and R-$BLDHs^{Cy7}$ intravenously via the tail vein when the tumors grew to about 1000 mm^3 in size. The engineered targeted nanomaterials were designed to circulate within the bloodstream and preferentially accumulate at the tumor site through ligand-receptor interactions. The emitted Cy7 signals were detected using in vivo imaging systems (IVIS), enabling real-time tracking of the nanomaterials' distribution. Imaging data were subsequently analyzed and quantified, including assessments of fluorescence intensity within the targeted region, quantification of nanomaterial accumulation, and comparisons with control or non-targeted groups. Mice were anesthetized and subjected to IVIS fluorescence imaging at 1, 6, 12, and 24 hours post-injection. Following the final imaging session, mice were euthanized, and the tumor, as well as key organs (kidneys, heart, spleen, lungs, and liver), were collected for comparative analysis of fluorescence accumulation levels.

Multiple Immunofluorescence Staining

Tumor samples were also harvested for immunofluorescence analysis to evaluate immune cell infiltration, focusing on dendritic cells (CD11c) and

cytotoxic T cells (CD3/CD8). Following fixation in 4% paraformaldehyde and embedding, tissue sections were deparaffinized, subjected to antigen retrieval, and blocked. Samples were then incubated with primary antibodies against CD11c, CD3, and CD8 (Proteintech). After secondary antibody staining, samples underwent cycle staining and sealing. Immune cell infiltration was examined using a fluorescence microscope.

Tumor Proteomics Analysis

Tumor proteomics analysis was performed on excised tumor tissues from nanomaterial-treated mice. Tumors were fixed in 4% paraformaldehyde, embedded in paraffin, and sectioned. Protein extraction from deparaffinized sections followed TCEP buffer treatment at 99°C. After trypsin digestion, peptides were desalted and analyzed via LC-MS/MS on a Q Exactive HF-X Hybrid Quadrupole-Orbitrap mass spectrometer. Proteins with differential expression were identified using the Wilcoxon paired signed-rank test, with fold changes > 2 and Benjamini-Hochberg adjusted p-values < 0.05 deemed significant. Enrichment analyses were conducted using KEGG and Gene Ontology (GO) databases, with p-values < 0.05 considered significant.

In vivo Lung Metastasis Tumor Model

To construct the lung metastatic tumor model, C57BL/6J mice were intravenously administered LLC cells at a dosage of 1 × 106 cells per mouse. Five days post-injection, the mice were randomly divided into four groups, and the therapeutic regimens were consistent with those described earlier. Fourteen days later, the mice were euthanized, followed by dissection and weighing of the lungs. The lung tissues were then fixed for hematoxylin-eosin (H&E) staining and immunofluorescent staining, which were used to conduct histological evaluation of the tumor nodules.

Biocompatibility

Fourteen days post-treatment, mice were euthanized, and their hearts, livers, spleens, lungs, kidneys, and tumors were harvested for histological analysis using H&E staining. Blood samples were collected to assess liver and kidney function, ensuring the biocompatibility of the nanomaterials.

Statistical Analysis

All statistical analyses were carried out using GraphPad Prism 9.0 and SPSS 20.0 software. Data are presented as means ± standard error of the mean (SEM) based on at least three independent experiments. Unpaired two-tailed t-tests were used to compare differences between groups, and a p-value of less than 0.05 was considered statistically significant.

2. Supporting Figures

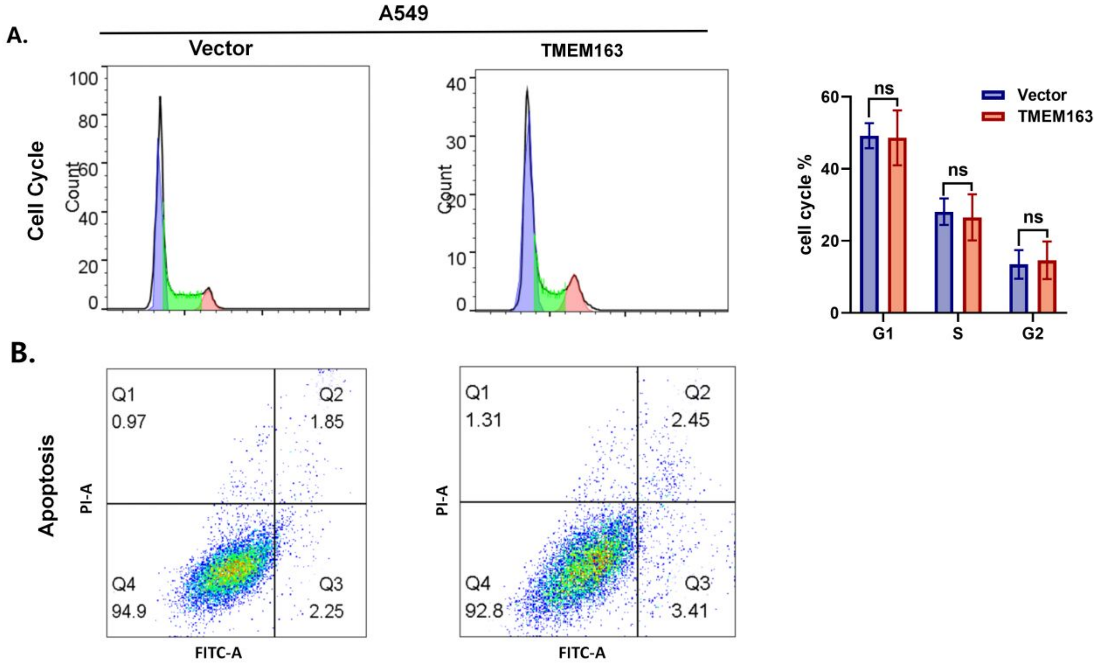

Figure S1. TMEM163 does not affect the cell cycle and apoptosis of lung cancer cells. A) Cell cycle assays of A549 cells of TMEM163 overexpression. **B)** Apoptosis assays of A549 cells of TMEM163 overexpression. Two-tailed student's t test was performed for statistical significance. Error bars represent mean ± SEM, ns, not significant.

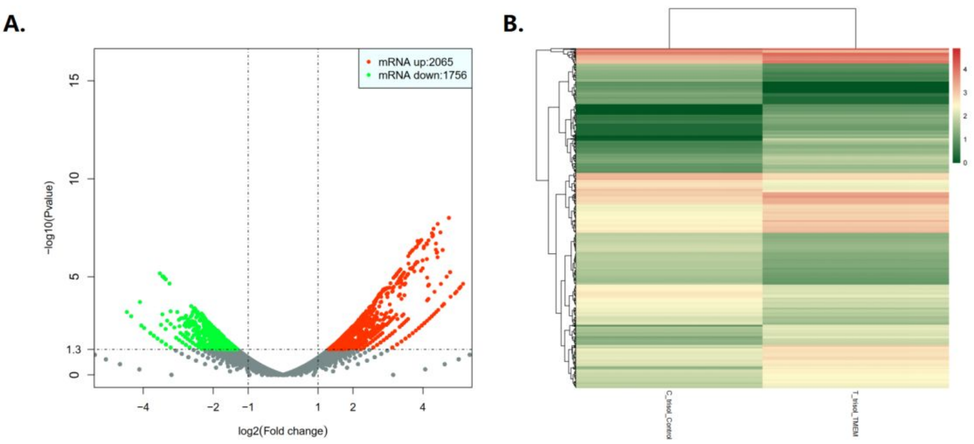

Figure S2. Volcano plot and heat map of differentially expressed genes.

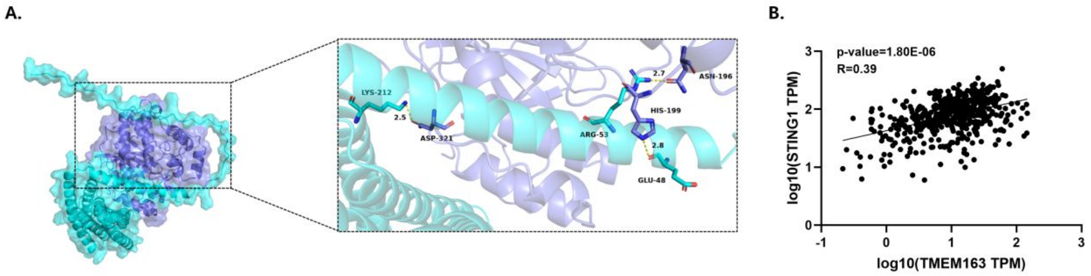

Figure S3. Prediction analysis of TMEM163 using relevant datasets. A) The strong interaction forces between MAPK14 and TMEM163. **B)** Correlation between TMEM163 TPM and STING1 TPM.

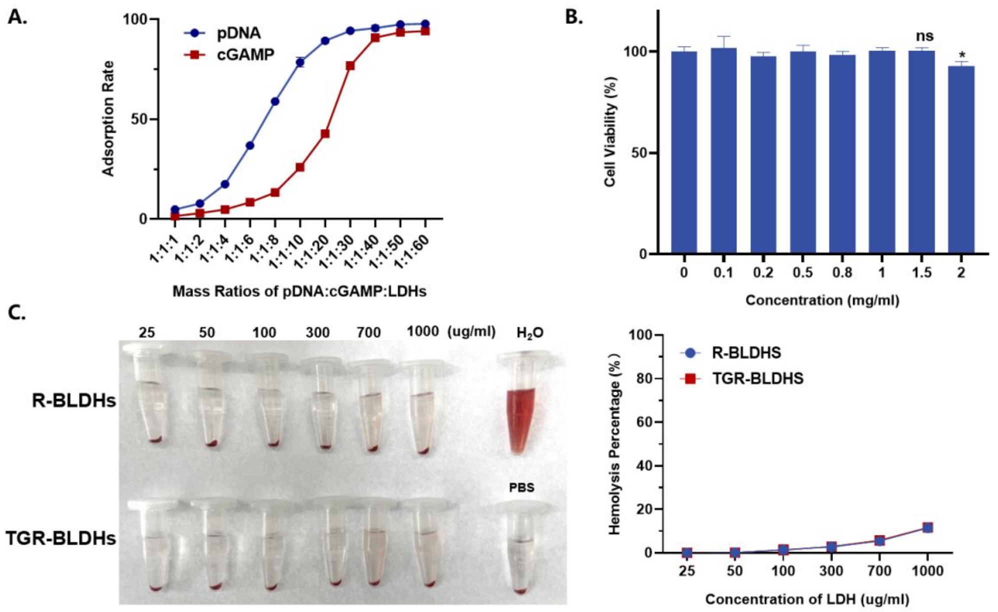

Figure S4. The high loading efficiency and biocompatibility of BSA/LDHs has been proven to be excellent. A) The assay of loading content of pDNA and cGAMP in BSA/LDHs. **B)** CCK-8 assay of BSA/LDHs with different concentration. **C)** Hemolysis test of BSA/LDHs with different concentration.

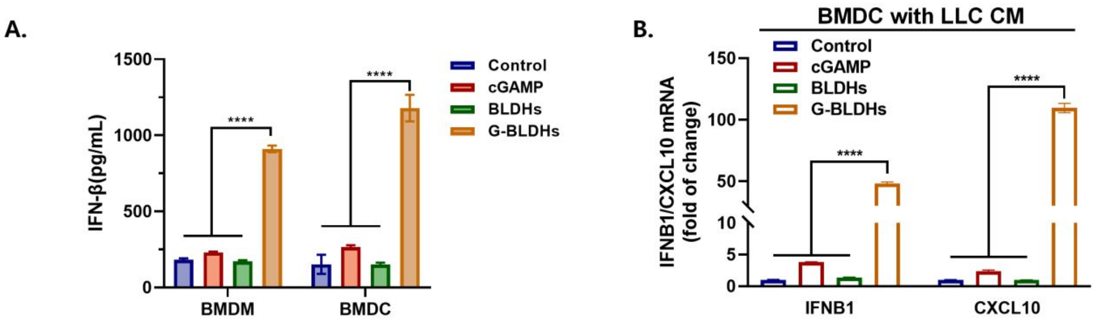

Figure S5. G-BLDHs nanomaterials promotes the cGAS/STING signaling transduction. A) The cytokine concentration in the culture medium of BMDM and BMDC cells with the indicated treatment was detected using an ELISA kit after 12 h of indicated treatment. **B)** The expression of IFNB1 and CXCL10 in BMDC cells incubated with CM from LLC cells after G-BLDHs treatments was detected using real-time PCR after 12 h of the indicated treatment.

A.

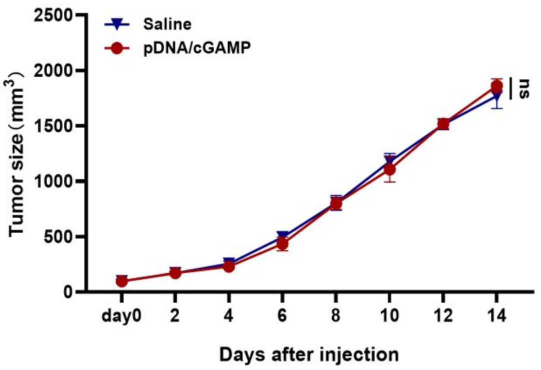

Figure S6. Therapeutic effect of free pTMEM163 and cGAMP on xenograft tumor models.

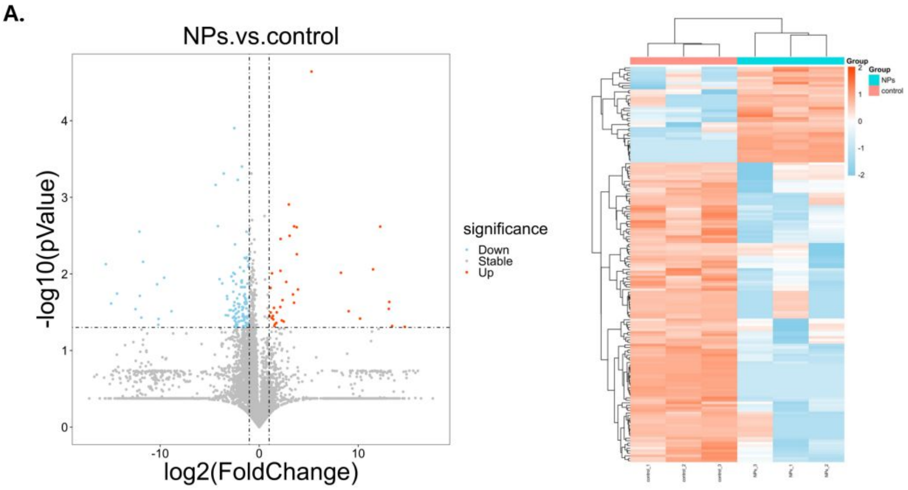

Figure S7. Volcano plot and clustering heat map of differential protein. Red indicates significantly up-regulated proteins, blue indicates significantly down-regulated proteins.

3. Supporting Tables

Supporting Table S1. List of RT-PCR primer sequences

Target	Forward primer (5' to 3')	Reverse primer (5' to 3')
TMEM163 (human)	TCCATCATTGTCACCCTGGC	CCGATCAGAACGCCTATGCT
18S (human)	GTAACCCGTTGAACCCCATT	CCATCCAATCGGTAGTAGCG
TMEM163 (mouse)	GGGTTGGAAGACCGAGGTTTA	GCCAGGGTGACAATAATGGACA
IFNB1 (mouse)	CAGCTCCAAGAAAGGACGAAC	GGCAGTGTAACTCTTCTGCAT
CXCL10 (mouse)	CCAAGTGCTGCCGTCATTTTC	GGCTCGCAGGGATGATTTCAA
β-Actin (mouse)	AACAGTCCGCCTAGAAGCAC	CGTTGACATCCGTAAAGACC

Supporting Table S2. Antibody

Antibody	Cat number	Dilution	Source	Company
Anti-TMEM163	ER1901-91	WB: 1:1000; IHC: 1:100	Rabbit	HuaBio
Anti-β-Actin	AF5003	WB: 1:1000	Rabbit	Beyotime
Anti-ERK1/2	sc-514302	WB: 1:1000	Mouse	Santa Cruz
Anti-p-ERK1/2	sc-136521	WB: 1:1000	Mouse	Santa Cruz
Anti-JNK1/2/3	AF1048	WB: 1:1000	Rabbit	Beyotime
Anti-p-JNK1/2/3	AF1762	WB: 1:1000	Rabbit	Beyotime
Anti-NF-κB	#8242	WB: 1:1000	Rabbit	Cell Signaling
Anti-p-NF-κB	#3033	WB: 1:1000	Rabbit	Cell Signaling
Anti-p38 MAPK	#9212	WB: 1:1000	Rabbit	Cell Signaling
Anti-p-p38 MAPK	#9211	WB: 1:1000	Rabbit	Cell Signaling
Anti-Ki67	AB2008	IF: 1:100	Rabbit	Beyotime
Anti-CD11c	117309	IF: 1:200	Mouse	Biolegend
Anti-CD3	100203	IF: 1:200	Mouse	Biolegend
Anti-CD8	140415	IF: 1:200	Mouse	Biolegend
